# Rab GTPase Mediating Regulation of NALP3 in Colorectal Cancer

**DOI:** 10.3390/molecules25204834

**Published:** 2020-10-20

**Authors:** Gülçin Tezcan, Ekaterina E. Garanina, Margarita N. Zhuravleva, Shaimaa Hamza, Albert A. Rizvanov, Svetlana F. Khaiboullina

**Affiliations:** 1 Institution of Fundamental Medicine and Biology, Kazan Federal University, 420008 Kazan, Russia; gulcintezcan@gmail.com (G.T.); kathryn.cherenkova@gmail.com (E.E.G.); k.i.t.t.1807@gmail.com (M.N.Z.); shaimaa.hamza@mail.ru (S.H.); rizvanov@gmail.com (A.A.R.); 2 Faculty of Dentistry, Department of Fundamental Sciences, Bursa Uludag University, Bursa 16240, Turkey; 3 Department of Microbiology and Immunology, University of Nevada, Reno, NV 89557, USA

**Keywords:** colorectal cancer, NALP3, inflammasome signalling, vesicle trafficking pathway, Rab GTPase

## Abstract

The NALP3 inflammasome signaling contributes to inflammation within tumor tissues. This inflammation may be promoted by the vesicle trafficking of inflammasome components and cytokines. Rab5, Rab7 and Rab11 regulate vesicle trafficking. However, the role of these proteins in the regulation of inflammasomes remains largely unknown. To elucidate the role of these Rab proteins in inflammasome regulation, HCT-116, a colorectal cancer (CRC) cell line expressing pDsRed-Rab5 wild type (WT), pDsRed-Rab5 dominant-negative (DN), pDsRed-Rab7 WT, pDsRed-Rab7 DN, pDsRed-Rab11 WT and pDsRed-Rab11 DN were treated with lipopolysaccharide (LPS)/nigericin. Inflammasome activation was analyzed by measuring the mRNA expression of *NLRP3*, *Pro-CASP1*, *RAB39A* and *Pro-IL-1β*, conducting immunofluorescence imaging and western blotting of caspase-1 and analysing the secretion levels of IL-1β using enzyme-linked immunosorbent assay (ELISA). The effects of Rabs on cytokine release were evaluated using MILLIPLEX MAP Human Cytokine/Chemokine Magnetic Bead Panel-Premixed 41 Plex. The findings showed that LPS/nigericin-treated cells expressing Rab5-WT indicated increased NALP3 expression and secretion of the IL-1β as compared to Rab5-DN cells. Caspase-1 was localized in the nucleus and cytosol of Rab5-WT cells but was localized in the cytosol in Rab5-DN cells. There were no any effects of Rab7 and Rab11 expression on the regulation of inflammasomes. Our results suggest that Rab5 may be a potential target for the regulation of NALP3 in the treatment of the CRC inflammation.

## 1. Introduction

Colorectal cancer (CRC) is the fourth most deadly cancer worldwide [1]. It appears that multiple stages of the CRC development are affected by inflammation [2]. Inflammation may be regulated by inflammasomes, intracellular multi-protein complexes [3] that consist of one or more inflammasome sensors, an adaptor, an apoptosis-associated speck-like protein containing a caspase recruitment domain (ASC) and caspase-1 [3,4]. NACHT, LRR and PYD domains-containing protein 3 (NALP3) is the most studied and well-characterized inflammasome [5]. NALP3 activation results in pro-caspase-1 cleavage and the release of an active form of caspase-1 [6,7]. Activated caspase-1 proteolytically cleaves inflammatory pro-IL-1β into its active form, IL-1β, establishing the inflammatory response [6,7]. The NALP3 inflammasome is required for the regulation of permeability and the regeneration of intestinal epithelium. However, the aberrant expression of NALP3 may result in severe intestinal inflammation, triggering the cyclical activation of the NALP3 inflammasome [8]. IL-1β and IL-18, the downstream signaling molecules from the NLRP3 pathway, have pleiotropic effects on CRC tumorigenesis [9,10,11,12]. Studies have indicated elevated expression of NALP3 inflammasome in CRC tumour tissue samples [13,14,15,16], and a positive correlation has been defined between NALP3 and distance metastasis [16]. However, several studies have also reported the effects of the low levels of the NALP3 inflammasome function on CRC development [17,18], which suggests that NALP3 inflammasome plays a balancing role in the regulation of inflammation [11]. 

Vesicle trafficking has been shown to promote inflammation using various mechanisms, such as the delivery of the inflammasome components (cytokines), the activation of surface receptors and the induction of the expression of inflammatory mediators [19,20]. However, our understanding of the molecular mechanisms of vesicle trafficking and its role in NALP3 inflammasome activation remains largely incomplete [21,22,23]. 

Rab proteins are part of the Ras-like small GTPase superfamily that controls vesicular trafficking [24,25]. Approximately 70 different Rab proteins have been identified in humans that are associated with a specific transport event [26]. Among these proteins, Rab39a, which is involved in late endocytosis [27,28], contains highly conserved caspase-1 cleavage sites, and the overexpression of Rab39a could result in increased IL-1β secretion [29]. Additionally, Rab8, which contributes to exocytosis regulation, has also been shown to facilitate IL-1β secretion [30,31]. Still, there are multiple Rab proteins whose role in the regulation of inflammasomes remains largely unknown. The main GTPase activated in early endocytosis is Rab5 [32,33,34,35]. When early endosomes mature into their late forms, Rab7 replaces Rab5 [32,33,34,35]. Rab11 also contributes to the recycling of cargo from early endosomes [35,36] and to exocytosis, together with Rab8 [37]. Whether these Rab- proteins contribute to inflammasome activation remains unknown.

In this study, we aim to investigate the role of Rab5, Rab7 and Rab11 in NALP3 inflammasome activation using an in-vitro model of CRC. We found that overexpression of Rab5 enhances inflammasome expression and the secretion of cytokines. In contrast, when Rab5 was inhibited, the expression of NALP3 reduced. We observed no significant effect of Rab7 and Rab11 overexpression on NALP3 inflammasome activation.

## 2. Results

**Figure 1:**
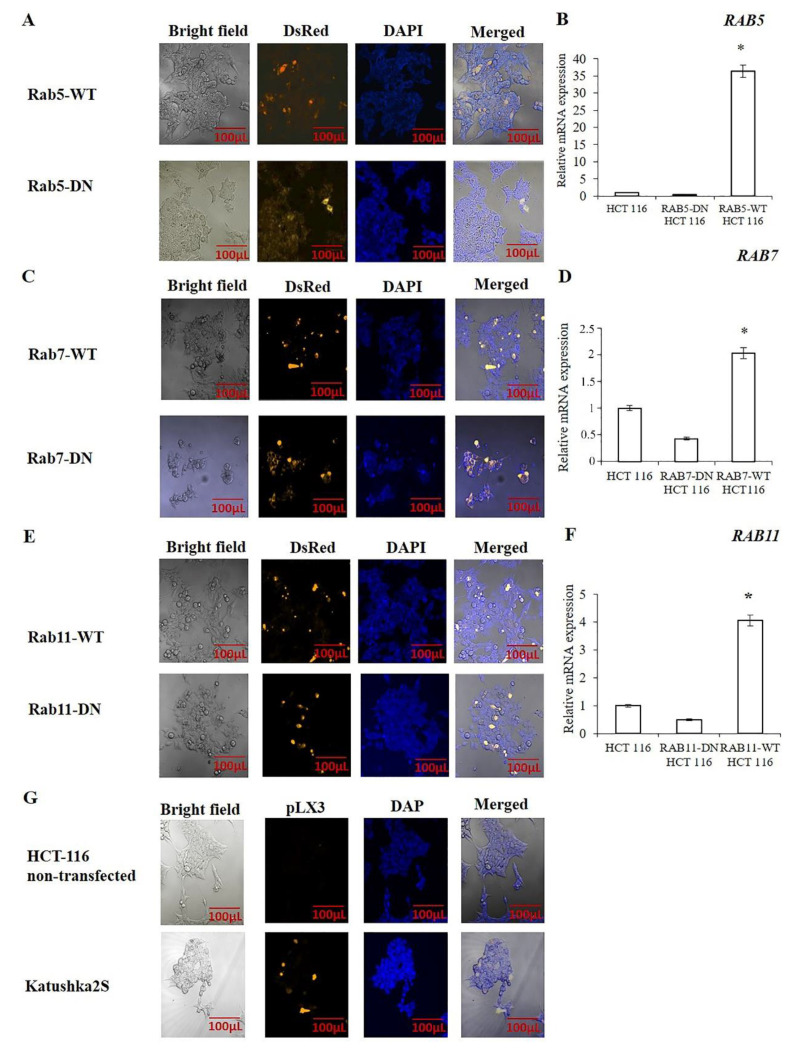
Expression of the Rab5, Rab7 and Rab11 in HCT-116 cells. Cells were transfected with pDsRed-rab5-WT, pDsRed-rab5-DN, pDsRed-rab7-WT, pDsRed-rab7-DN, pDsRed-rab11-WT and pDsRed-rab11-DN and protein expression was observed using 556 nm excitation wavelengths. (**A**) IF analysis of the cells transfected with pDsRed-rab5-WT and pDsRed-rab5-DN. (**B**) The changes in transcript level of *RAB5* in pDsRed-rab5-WT and pDsRed-rab5-DN transfected HCT116 cells. (**C**) IF analysis of cells transfected with pDsRed-rab7-WT and pDsRed-rab7-DN. (**D**) The changes in transcript level of *RAB7* in pDsRed-rab7-WT and pDsRed-rab7-DN transfected HCT116 cells. (**E**) IF analysis of cells transfected with pDsRed-rab11-WT and pDsRed-rab11-DN. (**F**) The changes in transcript level of *RAB11* in pDsRed-rab11-WT and pDsRed-rab11-DN transfected HCT116 cells. (**G**) IF analysis of cells transfected with pLX303-Katushka2S. (*n* = 3 for each experiment. In (**A**,**C**), **E**,**G**), transfection efficiency was measured using ImageJ software. In (**B**,**D**,**F**), * *p* value was calculated using One Way Anova and Tukey Test. * *p* value < 0.05.

The transiently transfected cell proportions with pDsRed-rab5-WT, pDsRed-rab5-DN, pDsRed-rab7-WT, pDsRed-rab7-DN, pDsRed-rab11-WT and pDsRed-rab11-DN are shown in Figure 1. The fusion rab-WT and rab-DN proteins expressed in HCT-116 cells (pDsRed-rab5-WT: 49.4 ± 1.9%; pDsRed-rab5-DN: 46.4 ± 1.6%; pDsRed-rab7-WT: 56.8 ± 1.2%, pDsRed-rab7-DN: 69.4 ± 1.7%; pDsRed-rab11-WT: 40.9 ± 2.7% and pDsRed-rab11-DN: 40.7 ± 1.6%). In order to observe the changes in the transcription level of *RAB5*, *RAB7* and *RAB11* in HCT-116 cells, caused by plasmid transfection, mRNA expression analyze was assessed for *RAB5*, *RAB7* and *RAB11*. After transfecting cells with WT-pDsRed-Rabs, the transcript level of *RAB5*, *RAB7* and *RAB11* were significantly increased as compared to control, whereas, transfection of DN-pDsRed-Rabs did not cause a significant change in the transcript level of *RAB5*, *RAB7* and *RAB11* in HCT-116 cells (Figure 1 and Supplementary Table S2). 

### 2.1. The Effects of Rab5 on NALP3 Inflammasome Activation

**Figure 2:**
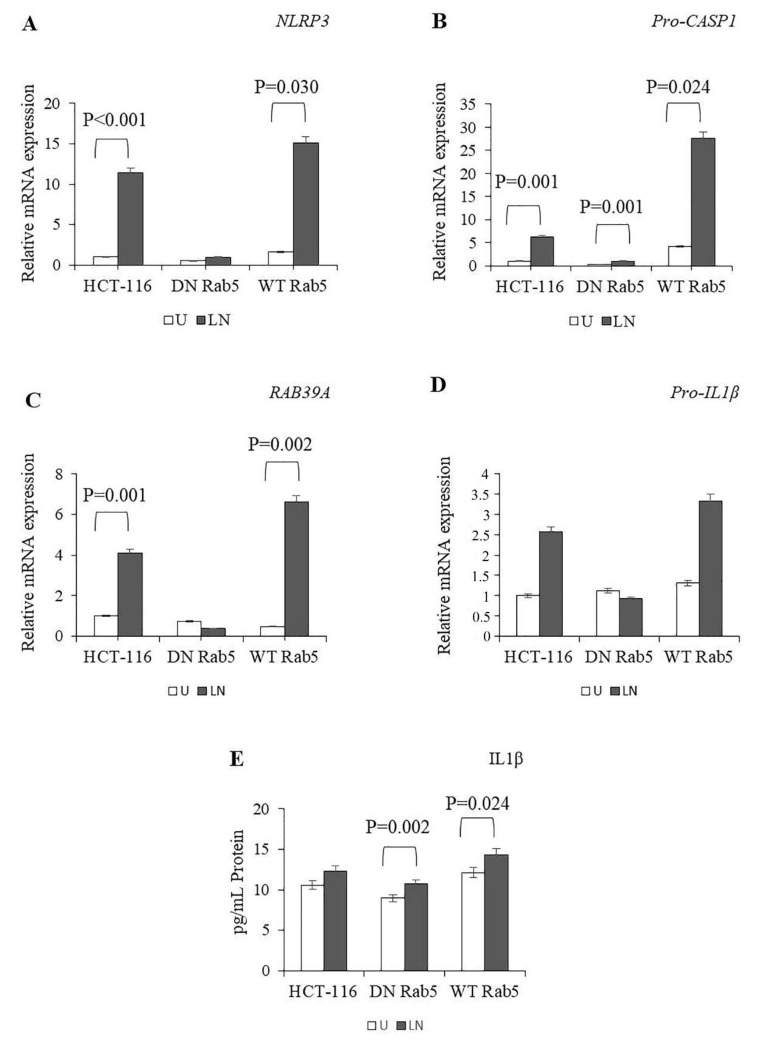
The effects of Rab5 on the NALP3 inflammasome. To assess NALP3 activation, HCT116 cells transfected with pDsRed-Rab5 WT and pDsRed-Rab5 DN were primed with LPS (1 µg/mL) for 3 hours and then treated with nigericin (20 µM) for 24 hours. (**A**–**D**) The effects of DN Rab5 and WT Rab5 on the expression of *NLRP3*, *RAB39A*, *Pro-CASP1* and *Pro-IL1β* in the untreated and LPS/nigericin-treated HCT116 cells. (**E**) The DN Rab5 and WT Rab5 effect on IL1β secretion in the untreated and LPS/nigericin-treated HCT116 cells. U: untreated; LN: LPS/nigericin treated (*n* = 3 for each experiment; *p*-values were calculated using independent sample T test).

NALP3 activation requires two signals: priming with LPS followed by activation using nigericin [38]. Therefore, we treated HCT-116 cells with LPS followed by nigericin and then analysed the expression of *NLRP3*, *Pro-CASP1*, *RAB39A* and *pro-IL-1β*. LPS/nigericin significantly increased the expression of *NLRP3*, *Pro-CASP1* and *RAB39A* (Supplementary Table S3). After WT and DN Rab5 transfections, LPS/nigericin significantly increased the expression of *Pro-CASP1*, while it had no effect on the expression of *NLRP3*, *RAB39A* and *pro-IL1β* in DN Rab5 cells compared to the control (Supplementary Table S4). In WT Rab5 cells, LPS/nigericin induced the expression of *NLRP3*, *Pro-CASP1* and *RAB39A* as compared to the control (Figure 2A–D; Supplementary Table S4). When we compared the NALP3 activation between the DN and WT Rab5 HCT116 cells, there was no difference in the expression of NALP3-associated genes, such as *NLRP3*, *Pro-CASP1*, *RAB39A* and *Pro-IL-1β*. However, LPS/nigericin induced the expression of *NLRP3* (*p* < 0.001), *Pro-CASP1* (*p* = 0.001), *RAB39A* (*p* < 0.0001) and *Pro-IL-1β* (*p* = 0.009) in WT Rab5 cells as compared to DN Rab5 cells (Supplementary Table S5). Collectively, our results showed that DN Rab5 and WT Rab5 did not affect NALP3-related gene expressions in untreated cells. However, after stimulation, levels of NALP3-related genes were higher in WT Rab5 cells compared to DN Rab5 expressed cells. 

NALP3 activated caspase-1 cleaves the precursors for pro-IL-1β to release mature cytokine [39]. These cytokines could promote an inflammatory form of the cell death named pyroptosis, which is linked to pore formation in the plasma membrane and the secretion of mature IL-1β [40]. Therefore, we analysed the effects of DN and WT Rab5 on the secretion of IL-1β in LPS/nigericin-treated HCT116 cells. Our results showed that in both DN and WT Rab5 cells, LPS/nigericin treatment significantly induced IL-1β secretion as compared to the controls (DN Rab5: 1.78pg/mL; WT Rab5: 2.23 pg/mL as compare to controls; Figure 2E). When we analysed the IL-1β secretion between the DN and WT Rab5 cells, we found that WT Rab5 significantly increased IL1β secretion when treated with LPS/nigericin as compared to DN Rab5 (Figure 2E; Supplementary Table S6). Therefore, our findings showed that in WT Rab5 cells, NALP3-dependant secretion of IL-1β was significantly higher than that in DN Rab5 cells.

**Figure 3:**
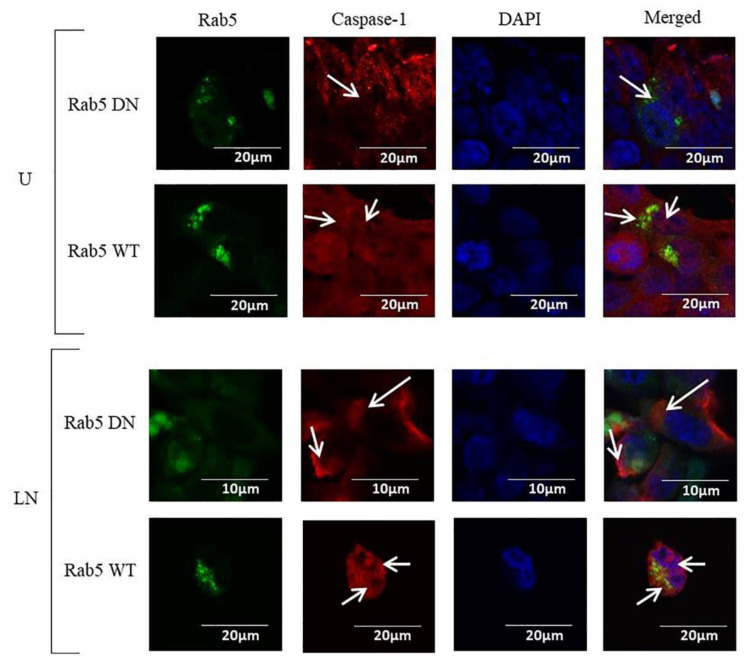
The effects of Rab5 DN and Rab5 WT expression on caspase-1 localization. Nigericin (20 µM) treatment for 24 h and three hours of pre-incubation with LPS (1 µg/mL) were used to activate the NALP3 inflammasome after the transfection of pDsRed-rab5-WT and pDsRed-rab5-DN to the HCT116 cells. Caspase-1 was found only in the cytosol in the control and LPS/nigericin-treated DN Rab5 cells, while it was localized in the nuclei and cytosol in the WT Rab5 cells. Arrows show the localization of caspase-1. DN and WT Rab5 proteins were visualized using green coloring with 488 nm excitation wavelengths. Caspase-1 was visualized using a 590 nm excitation wavelength and red coloring. The nucleus was visualized using DAPI. U: untreated; LN: LPS/nigericin, DN: dominant negative; WT: wild type (*n* = 3 for each experiment).

The NALP3 inflammasome recruits pro-caspase-1 and activates caspase-1, which cleaves the IL-1β precursor into its mature IL-1β form [31]. Therefore, we sought to determine the effects of Rab5 on the expression of caspase-1. Our results indicated that in DN Rab5 transfected cells, caspase-1 was found only in the cytosol in the control and LPS/nigericin-treated cells; however, in the WT Rab5 cells, caspase-1 was localized in the nuclei and cytosol (Figure 3). 

### 2.2. The Effects of Rab7 on NALP3 Inflammasome Activation

**Figure 4:**
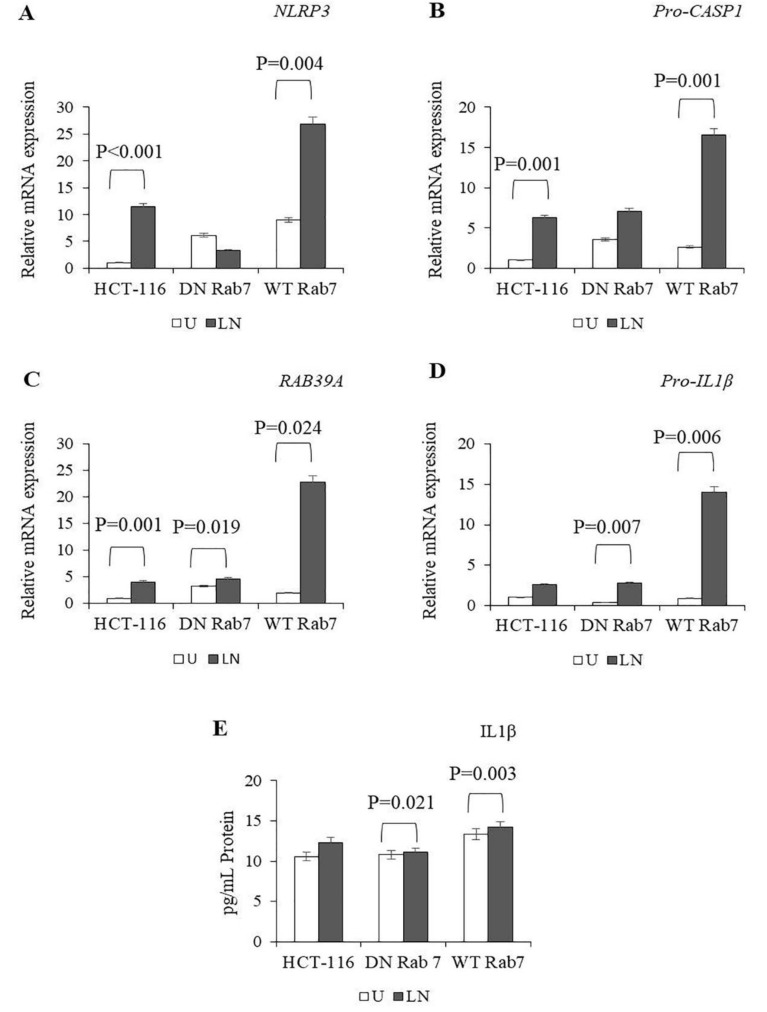
The effects of Rab7 on NALP3 inflammasome activation. To assess NALP3 activation, HCT116 cells transfected with pDsRed-Rab7 WT and pDsRed-Rab7 DN were primed with LPS (1 µg/mL) for three hours and then treated with nigericin (20 µM) for 24 h. (**A**–**D**) The effects of DN Rab7 and WT Rab7 on the expression of *NLRP3*, *RAB39A*, *Pro-CASP1* and *Pro-IL1β* in the untreated and LPS/nigericin-treated HCT116 cells. (**E**) The effects of DN Rab7 and WT Rab7 on IL1β secretion in the untreated and LPS/nigericin-treated HCT116 cells. U: untreated; LN: LPS/nigericin-treated (*n* = 3 for each experiment; *p*-values were calculated using independent sample T test).

In DN Rab7 cells, LPS/nigericin increased the expression of *RAB39A* and *Pro-IL-1β*, whereas in WT Rab7 cells, LPS/nigericin induced the expression of *NLRP3*, *Pro-CASP1, RAB39A* and *pro-IL1β* compared to the control (Figure 4A–D; Supplementary Table S7). When we compared the levels of NALP3 activation between the DN and WT Rab7 expressed HCT116 cells, there was no difference in the expression of NALP3-related genes, such as *NLRP3*, *Pro-CASP1*, *RAB39A* and *Pro-IL-1β*. However, after LPS/nigericin treatment, the expression levels of *NLRP3* (*p* < 0.001), *Pro-CASP1* (*p* = 0.003), *RAB39A* (*p* = 0.006) and *pro-IL-1β* (*p* = 0.002) were higher in the WT Rab7 cells compared to the DN Rab7 cells (Supplementary Table S8). Our findings showed that after the stimulation of NALP3, the expression of NALP3-related genes was higher in the WT Rab7 cells compared to the DN Rab7 cells.

LPS/nigericin significantly increased the secretion of IL-1β in DN Rab7 and WT Rab7 cells compared to the controls. There were no statistically significant differences in IL-1β secretion between the DN and WT Rab7 cells after LPS/nigericin treatment (Figure 4E; Supplementary Table S9). Collectively, our findings showed that although DN and WT Rab7 play roles in the regulation of NALP3-related genes, Rab7-dependent regulation of NALP3-related genes does not statistically correlate with IL-1β secretion. 

**Figure 5:**
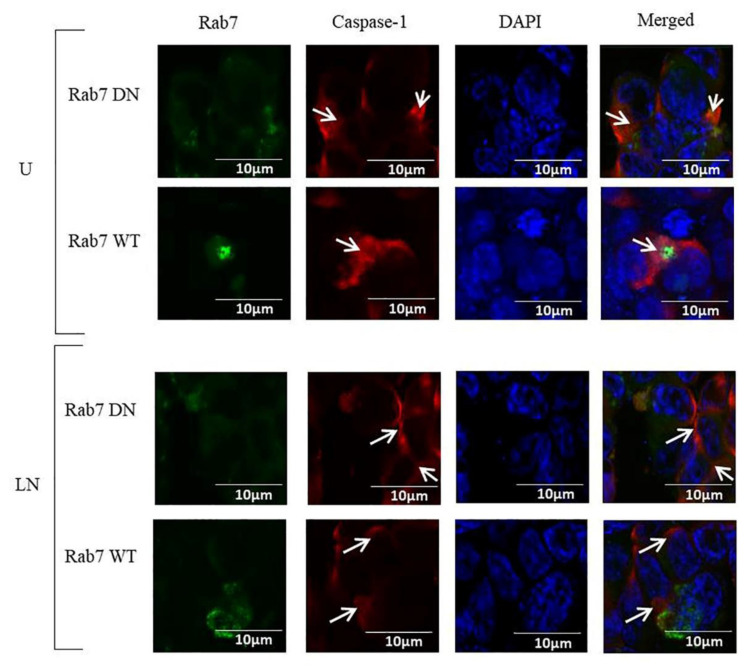
The effects of the transfection of Rab7 DN and Rab7 WT plasmids on the localization of caspase-1. Nigericin (20 µM) treatment for 24 h and three hours of pre-incubation with LPS (1 µg/mL) were used to activate the NALP3 inflammasome after the transfection of pDsRed-rab7-WT and pDsRed-rab7-DN to the HCT116 cells. Caspase-1 was found only in the cytosol in the control and LPS/nigericin-treated DN Rab7 and WT Rab7 cells. Arrows show the localization of caspase-1 DN and WT Rab7. Proteins were visualized using green coloring with 488 nm excitation wavelengths. Caspase-1 was visualized using a 590 nm excitation wavelength and red coloring. The nucleus was visualized using DAPI. U: untreated; LN: LPS/nigericin; DN: dominant negative; WT: wild type (*n* = 3 for each experiment).

Caspase-1 was localized in the cytosol of DN Rab7 HCT116 cells and in the cytosol and nucleus of WT Rab7 HCT116 cells. In addition, Activation of NALP3 with LPS/nigericin did not affect the expression and localization of caspase-1 (Figure 5). 

### 2.3. The Effects of Rab11 on NALP3 Inflammasome Activation

**Figure 6:**
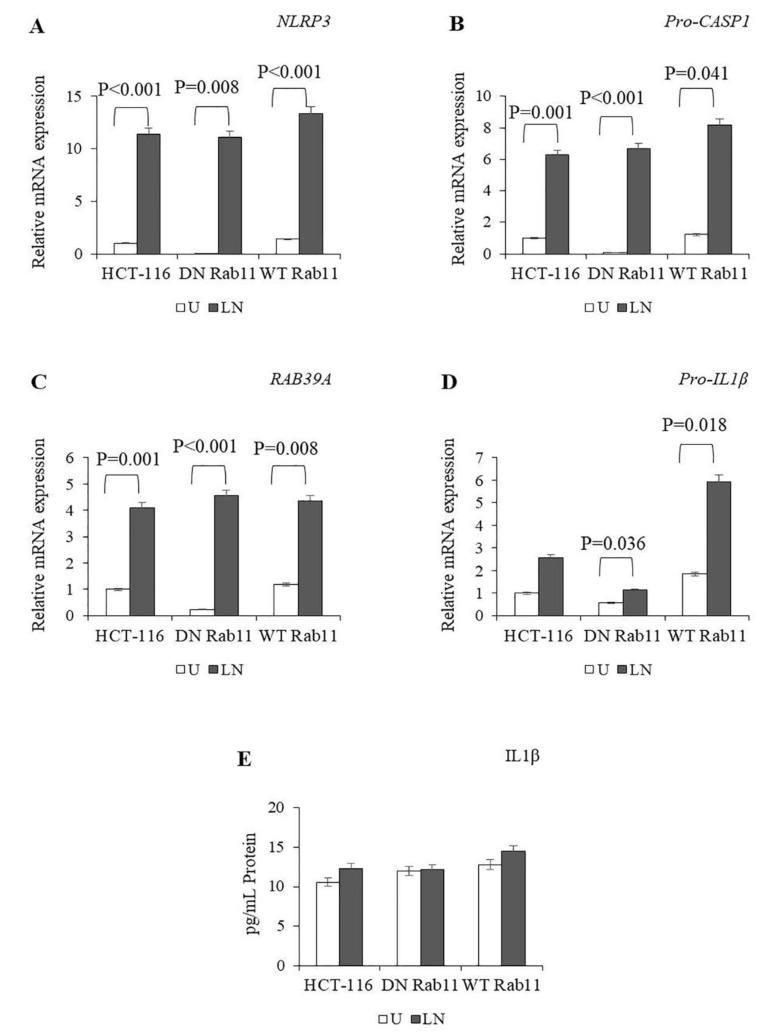
The effects of Rab11 on NALP3 inflammasome activation. To assess NALP3 activation, HCT116 cells transfected with pDsRed-Rab11 WT and pDsRed-Rab11 DN were primed with LPS (1 µg/mL) for three hours and then treated using nigericin (20 µM) for 24 h. (**A**–**D**) The effects of DN Rab11 and WT Rab11 on the expression of *NLRP3*, *RAB39A*, *Pro-CASP1* and *Pro-IL1β* in the control and LPS/nigericin-treated cells. (**E**) The effects of DN Rab11 and WT Rab11 on IL1β secretion in the control and LPS/nigericin-treated cells. U: untreated; LN: LPS/nigericin-treated. (*n* = 3 for each experiment; *p*-values were calculated using independent sample *T* test).

In the untreated WT Rab11 cells, the expression of *NLRP3*, *Pro-CASP1*, *RAB39A* and *Pro-IL-1β* was higher compared to the untreated DN Rab11 cells (Figure 6A–D; Supplementary Table S10). The DN and WT Rab 11 cells increased the expression of *NLRP3*, *Pro-CASP1*, *RAB39A* and *pro-IL1β* after treatment with LPS/nigericin compared to the control (Supplementary Table S10). The expression of *Pro-CASP1* (*p* = 0.022), *RAB39A* (*p* = 0.040) and *Pro-IL-1β* (*p* = 0.005) was higher in WT Rab11 cells compared to DN Rab11 cells after LPS/nigericin treatment (Supplementary Table S11).

LPS/nigericin did not affect the secretion of IL-1β in the DN and WT Rab11 cells compared to the control (Figure 6E; Supplementary Table S12). Collectively, our findings suggest that DN and WT Rab11 make only a limited contribution to the regulation of NALP3 and the secretion of maturated IL-1β. 

**Figure 7:**
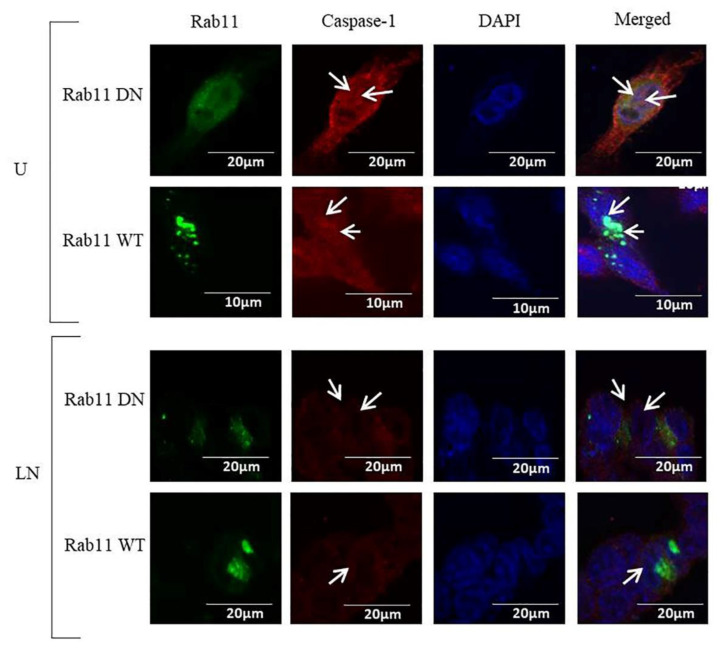
The effects of DN Rab11 and WT Rab11 on the localization of caspase-1 in HCT116 cells. Nigericin (20 µM) treatment for 24 h and three hours of pre-incubation with LPS (1 µg/mL) were used to activate the NALP3 inflammasome after the transfection of pDsRed-rab11-WT and pDsRed-rab11-DN to the HCT116 cells. Caspase-1 was found only in the nuclei and cytosol in the control and LPS/nigericin-treated DN Rab11 and WT Rab11 cells. Arrows show the localisation of caspase-1. DN and WT Rab11 proteins were visualized using green coloring with a 488 nm excitation wavelength. Caspase-1 was visualized using a 590 nm excitation wavelength and red coloring. The nucleus was visualized using DAPI. U: untreated; LN: LPS/nigericin; DN: dominant negative; WT: wild type (*n* = 3 for each experiment).

Caspase-1 was localized in the nuclei and cytosol of WT Rab11 and in the cytosol of DN Rab11 HCT116 cells. NALP3 activation using LPS/nigericin did not affect the localization of caspase-1 (Figure 7). 

### 2.4. The Effects of Rab5, Rab7 and Rab11 on Pro-Caspase-1 Expression and Cleavage

**Figure 8:**
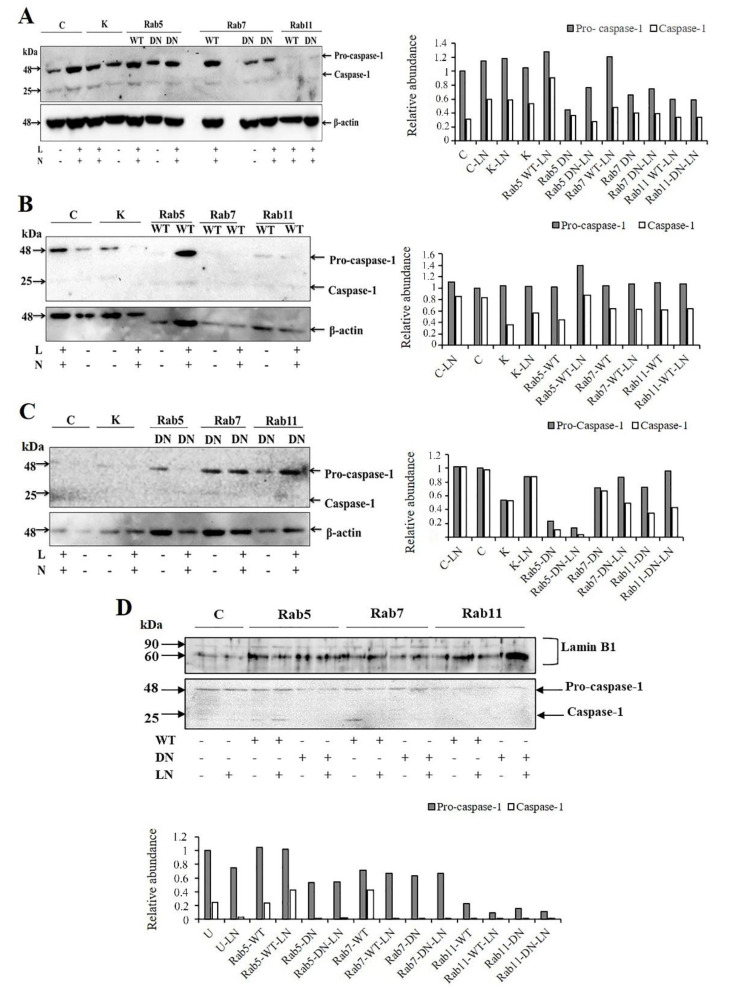
The effects of LPS/nigericin treatment on the expression of pro-caspase-1 and caspase-1 in Rab5-WT, Rab5-DN, Rab7-WT, Rab7-DN, Rab11-WT and Rab11DN cells. Nigericin (20 µM) treatment for 24 h and three hours of pre-incubation with LPS (1 µg/mL) were used to activate the NALP3 inflammasome after the transfection of pDsRed-rab5-WT, pDsRed-rab5-DN, pDsRed-rab7-WT, pDsRed-rab7-DN, pDsRed-rab11-WT and pDsRed-rab11-DN to the HCT116 cells. (**A**) Whole cell lysates were immunoblotted with caspase 1 and β-actin antibodies. (**B**) Cytosolic fractions of Rab5-WT, Rab7-WT and Rab11-WT cells were immunoblotted with caspase 1 and β-actin antibodies. (**C**) Cytosolic fractions of Rab5-DN, Rab7-DN and Rab11-DN cells were immunoblotted with caspase 1 and β-actin antibodies. (**D**) Nuclear fractions of Rab5-WT, Rab5-DN, Rab7-WT, Rab7-DN, Rab11 WT and Rab11-DN cells were immunoblotted with caspase 1 and Lamin B antibodies. The density of protein bands was measured using ImageJ software. C: Non-transfected HCT-116, control cells, K: Katushka2S transfected cells; WT: wild type; DN: dominant negative; L: LPS; N: nigericin; LN: LPS/nigericin.

The effects of LPS/nigericin on caspase-1 protein expression in cells transfected with WT and DN Rab5, Rab7 and Rab11 were demonstrated using western blot (Figure 8A). LPS/nigericin increased the expression of pro-caspase 1 and active caspase-1 as compared to untreated HCT-116 cells. In addition, the activation of caspase-1 was higher in Rab5-WT-LN cells compared to Rab5-DN-LN cells (Figure 8A). In the Rab5-WT cells, caspase-1 was localized in the nuclei and cytosol, whereas a decreased expression of caspase-1 was observed in the nuclei and cytosol of the Rab5-DN infected cells (Figure 8B–D). In cells transfected with Rab7-WT cells, the expression of pro-caspase-1 and active caspase-1 were higher as compared to the Rab-7-DN cells. Caspase-1 was expressed in the cytosol and there was no caspase-1 expression in nuclear fraction of Rab7-DN cells whereas an expression of caspase-1 was observed in nuclear fraction of Rab7-WT cells. Pro-caspase-1 expression was observed in the nuclei and cytosol of Rab11-WT and Rab11-DN cells. However, caspase-1 was expressed only in cytosol and was not detected in nucleus of both forms of Rab11 (Figure 8B–D). 

### 2.5. The Effects of Rab5 on Cytokine Secretion

**Figure 9:**
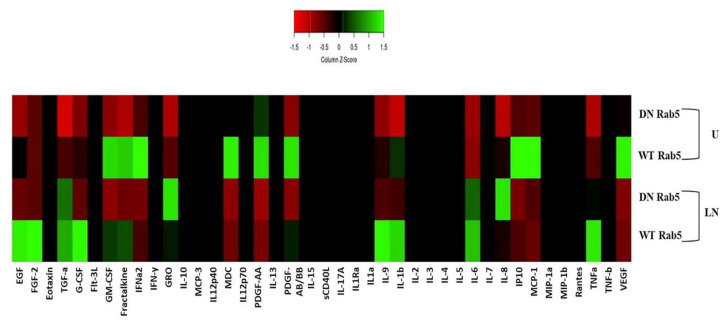
Cytokine secretion pattern in WT Rab5 and DN Rab5 expressed HCT116 cells after NALP3 activation. Nigericin (20 µM) treatment for 24 h and three hours of pre-incubation with LPS (1 µg/mL) was used to activate the NALP3 inflammasome after the transfection of pDsRed-rab5-WT and pDsRed-rab5-DN to the HCT116 cells. U: untreated; LN: LPS/nigericin; DN: dominant negative; WT: wild type (*n* = 1 for each experiment).

Cell culture media from the WT and DN Rab5 HCT116 cells were collected 24 h after the LPS/nigericin treatment and used to analyse the release of cytokines. WT Rab5 affected the levels of cytokines secreted by tumour cells upon LPS/nigericin treatment (Figure 9). Cytokine levels in the media from the HCT116 cells expressing DN and WT Rab5 followed by treatment with LPS/nigericin are summarized in Supplementary Table S13. 

The HCT116 cells expressing WT and DN Rab5 were secreting high levels of EGF, TGF-a, G-CSF, fractalkine, IL-9, IL-1β, IL-6 and TNFα after LPS/nigericin treatment compared to the controls. However, the levels of these cytokines were higher in the WT Rab5 cells compared to the DN Rab5 cells. Interestingly, although LPS/nigericin treatment decreased the release of IFN-a2, CCL-22 (MDC) and PDGF-AA in the DN Rab5 cells compared to the DN Rab5 controls, these cytokine levels increased in LPS/nigericin-treated WT Rab5 cells compared to untreated WT Rab5 cells and DN Rab5 cells treated with LPS/nigericin.

## 3. Discussion

Low-level inflammation appears to be essential to establishing the balance between the immune system and the highly antigenic environment in the gastrointestinal system to maintain homeostasis in health. However, failure in this balance may lead to the chronic intestinal inflammation that is fundamental in the development of the CRC [41]. The NALP3 signalling was identified as one of the underlying molecular mechanisms of chronic intestinal inflammation, leading to persistent overproduction of pro-inflammatory cytokines, including IL-1β. These cytokines may promote aberrant intestinal epithelial cell proliferation, survival and angiogenesis and lead to epithelial dysplasia and the development of CRC [41,42]. IL-1β can be released from cells in two distinct ways: Gasdermin D plasma membrane pore formation and the unconventional pathway dependant on caspase-1 activation [43]. Vesicular transport, including endosomes and exosomes, plays an active role in the delivery of inflammasome components and cytokines [19,20]. This vesicular transport may be regulated by Rab GTPase proteins [44]. In this study, we analysed the role of Rab5 and Rab7 proteins, which regulate the early and late endosome, as well as Rab11, which is part of the recycling pathway in the early endosome [35,36] and exocytosis [37], on the regulation of the NALP3 inflammasome in a CRC cell line, HCT116. 

NALP3 inflammasome activation requires two signals as follows: priming and activation [45]. LPS is commonly used as a priming signal [46], while the second signal could include multiple stimuli, including ATP and potassium efflux agents [47]. Nigericin, a microbial toxin, is a potassium ionophore and is often used as a second signal for NALP3 activation [48,49]. Therefore, we used LPS and Nigericin as two signals for NALP3 activation in cells expressing WT and DN forms of Rab5, Rab7 and Rab11. 

Inflammasome activation requires endocytosis [50], which involves Rab5 endosomal GTPase expressed on early endocytic vesicles [51,52]. The activation of Rab5 results in the recruitment of specific proteins and the synthesis of specific lipids on endosomes [35]. Our results have demonstrated that DN Rab5 induces the expression of *Pro-CASP1* and having limited effects on the expression of other NALP3-associated genes, including *NLRP3*, *RAB39A* and *Pro-IL-1β* in cells treated with LPS/nigericin compared to controls. However, WT Rab5 enhances the expression of NALP3 and associated genes in cells treated with LPS/nigericin compared to control and LPS/Nigericin-treated DN Rab5 cells. Therefore, our findings suggest that WT Rab5 enhances the expression of NALP3 and associated genes in cells treated with LPS/nigericin. However, DN Rab5 has a limited effect on NALP3 expression. 

In DN Rab5 cells, caspase-1 was localized only in the cytosol, while in WT Rab5 cells, it was localized in the nuclei and cytosol after NALP3 activation. The inactive form of pro-caspase-1 is known to contain nuclear localization signals [53], while inflammasome assembly occurs in the cytosol [54]. Therefore, our findings suggest that the expression of DN Rab5 leads only to enhanced proteolytic activity of caspase-1. However, expression of WT Rab5 may upregulate pro-caspase-1 expression in the nucleus and enhances the proteolytic activity of caspase-1 in the cytosol, where inflammasomes are assembled to convert inactive pro-caspase-1 to an active form [55]. Supporting these findings, we have demonstrated increased secretion of IL-1β in WT Rab5 cells compared to DN Rab5 cells after nigericin and LPS/nigericin treatments. Collectively, our results have demonstrated that WT Rab5 enhances NALP3 activation, the cytosolic expression of caspase-1 and the production and secretion of IL-1β in CRC. 

Vesicle trafficking plays a role in the delivery of inflammasome components and cytokines, as well as inducing the expression of inflammatory mediators [19,20]. We have demonstrated that LPS/nigericin treatment may induce the secretion of EGF, TGF-a, G-CSF, fractalkine, IL-9, IL-1β, IL-6 and TNFα in both WT and DN Rab5 HCT116 cells. These cytokines may promote formation of inflammatory microenvironments and contribute to the aggressiveness of tumours [56,57,58,59,60,61,62,63,64]. In our study, the secretion of these cytokines was higher in WT Rab5 cells compared to DN Rab5 cells. Therefore, our data suggest that WT Rab5 may promote the secretion of inflammatory cytokines in NALP3-activated CRC. It should be noted that, the cytokine assay was carried out in a single sample for each group. Therefore, statistical analysis in an increased number of experiment repeat will clarify the significantly affected cytokines and the significancy of changes in their secretion levels.

During endosomal maturation and remodelling, Rab5 promotes the GTP loading of Rab7 [32,33,34,35]. The Rab5-to-Rab7 cascade is required not only in endosomal development but also for autophagosome formation [65]. Interestingly, autophagosomes were shown to negatively regulate inflammasome signalling to prevent excessive inflammation [30,66,67,68]. In our study, there was no significant difference in NALP3 expression between untreated DN and WT Rab7 cells. However, after LPS/nigericin exposure, the expression of the NALP3-related genes (*NLRP3*, *Pro-CASP1*, *RAB39A* and *pro-IL-1β)* was higher in WT Rab7 cells compared to DN Rab7 cells. On the other hand, LPS/nigericin treatment led to the secretion of IL-1β from both DN and WT Rab7 cells, and there was no significant difference in the levels of this cytokine secretion. Additionally, caspase-1 was expressed in the cytosol of the DN and WT Rab7 untreated controls and the NALP3-activated cells, suggesting that although WT Rab7 expression enhances the expression of NALP3, both DN and WT Rab7 cells can produce functionally active caspase-1 in the cytosol with the subsequent secretion of IL-1β [55]. Supporting this assumption, we have found that Rab7 expression did not affect the secretion of IL-1β in untreated and NALP3-activated cells. Therefore, our data suggest that Rab7 plays a limited role in the regulation of the NALP3 inflammasome and the processing and secretion of IL-1β.

Rab11 is associated with perinuclear recycling endosomes and endocytosed proteins, as well as participating in the exocytosis of extracellular vesicles (EV) [69]. Studies have demonstrated that EV secretion is one of the most common outcomes of inflammasome activity [7]. Multiple NALP3 activators, such as ATP [22], monosodium urate and β-glucans [21,70] and Nigericin [7,71], can induce EV secretion by changing the transmembrane ionic balance of calcium and potassium, destabilizing the lysosome and activating caspase-1 [7]. Recently, the role of Rab11-family interacting protein 2 (FIP2) on NALP3 inflammasome activation has been demonstrated [72]. In our study, after LPS/nigericin treatments, both DN and WT Rab11 cells induced the mRNA expression of *NLRP3*, *RAB39A*, *Pro-CASP1* and *Pro-IL-1β* compared to the control. We also identified that LPS/nigericin enhanced the expression of *Pro-CASP1*, *RAB39A* and *Pro-IL-1β* in WT Rab11 cells. However, there were no differences in the secretion of IL-1β in the untreated and LPS/nigericin-treated DN and WT Rab11 cells. This could be because Rab11 interfered protein translation of pro-caspase-1 and maturation of the protein after the LPS/nigericin treatment. It was shown that, Rab11 has allosteric binding sites that could allow potential effector molecules to bind to a site other than the enzyme’s active site [73]. Therefore, we suggest that the effects of NALP3 activation by nigericin on the allosteric binding sites of Rab11 require further investigation. 

In conclusion, we have, to our knowledge, for the first time, elucidated the role of Rab5, Rab7 and Rab11 expression in NALP3 activation in CRC. We have found that LPS/nigericin treatment for WT Rab5 may enhance NALP3 activation and increase the secretion of inflammatory cytokines. Also, although WT Rab7 and Rab11 enhance the expression of NALP3-related genes, these Rabs do not affect NALP3 inflammasome activation. Our data show the role of Rab5 in inflammasome activation, suggesting that this GTPase could be potential therapeutic target for the suppression of inflammation in CRC. Regardless, confirmation of our findings in other CRC cell lines is required. In addition, it should be noted that the overexpression of WT and DN Rabs is a blunt tool given that many Rabs share effector and accessory proteins and their overexpression could sequester them away from other endogenous Rabs that may also be involved in inflammasome activation [74]. Therefore, knocking down these Rabs using RNAi or CRISPR in future research will likely to confirm our primary findings about their function in NALP3 inflammasome activation. 

## 4. Material and Methods

### 4.1. Cell Line Maintenance

The CRC cell line HCT116 (ATCC CCL-247) was purchased from the American Type Culture Collection (ATCC, Rockville, MD, USA). Cells were maintained in Dulbecco’s Modified Eagle Medium/Nutrient Mixture F-12 (DMEM/F-12) supplemented with 10% fatal bovine serum (FBS, Atlanta Biologicals), 2 mM L-glutamine, 25 U/mL penicillin, and 25 μg/mL streptomycin. Cells were grown at 37 °C in a humidified chamber supplemented with 5% CO_2_. 

### 4.2. Genetic Modification of Cells

In this study, we utilized the pDsRed-Rab5 wild type (WT), pDsRed-Rab5 dominant-negative (DN), pDsRed-Rab7 WT, pDsRed-Rab7 DN, pDsRed-Rab11 WT and pDsRed-Rab11 DN (Addgene, Cambridge, MA, USA) plasmid constructs to transfect HCT116 cells using the TurboFect transfection reagent (Thermo Fisher Scientific Inc., USA), according to the manufacturer’s protocol [75]. We used the pLX303-Katushka2S plasmid (Addgene, Cambridge, MA, USA) as a positive control for transfection efficiency. Transfection efficiency was evaluated using confocal microscopy. 

### 4.3. NALP3 Activation 

To assess the NALP3 activation, plasmid transfected HCT116 cells were primed with LPS (1 µg/mL, Sigma, St. Louis, MO, USA) for three hours, followed by Nigericin (20 µM, Invivogen, San Diego, CA, USA) treatment for 24 h. 

### 4.4. Real-Time qPCR

The total RNA was extracted using TRIzol (Sigma, St. Louis, MO, USA), as described previously [76]. The cDNA was generated using 500 ng of the total RNA and the RevertAid First Strand cDNA Synthesis Kit (Thermo Fisher Scientific, Inc., Waltham, MA, USA). The endogenous mRNA expression of the RAB5, RAB7 and RAB11 was analyzed through RT-qPCR using the primers summarized in Supplementary Table S1. The qPCR was carried out in a 10 µL reaction mixture (200 ng of cDNA and 10 μM each of the primer and qPCRmix-HS SYBR (Evrogen JSC, Moscow, Russia). The cycle parameters were as follows: 95 °C for 10 min and 45 cycles at 95 °C for 15 s and 60 °C for 60 s, followed by melting curve analyses in the CFX384 Touch™ Real-Time PCR Detection System (Biorad, CA, USA). The copy numbers in the sample and the Ct value for gene expression were determined using the CFX384 Touch™ Real-Time PCR Detection System software (Biorad, CA, USA). The 2^−ΔCt^ method was used to calculate the fold change in gene expression.

### 4.5. Enzyme-Linked Immunosorbent Assay (ELISA) 

IL-1β levels were determined in cell-free supernatants using commercially available ELISA kits (VECTOR-BEST, Novosibirsk, Russia). Experiments were performed in triplicate for each sample.

### 4.6. Immunofluorescence Analysis (IF)

The cells were fixed using 3.7% paraformaldehyde for 10 minutes at room temperature. WT and DN Rabs were detected using 556 nm excitation wavelengths in orange color [77]. Caspase-1 expression was analyzed using primary mouse monoclonal anti-caspase-1 antibody (sc-392736; Santa Cruz Biotechnology, 1:300) and secondary Alexa Fluor^®^ 594 Goat Anti-Mouse IgG antibody (A-11005, Invitrogen, 1:1000). To demonstrate proteins localizations, 488 nm excitation wavelength in green color was used for Rabs [78,79], while 590 nm excitation wavelengths in red color were used for Caspase-1. The nucleus was visualized using DAPI (D1306, Termofisher, 300 nM). Images were acquired using confocal laser scanning microscopy on an LSM 700 (Zeiss); ZEN 3.0 Black was used for image processing (Zeiss). The percentages of fusion protein-expressing cells were quantified using NIH ImageJ software version 1.52a.

### 4.7. Subcellular Fractionation

Cells were lysed in 500 μL of fractionation buffer (20 mM HEPES (pH: 7.4), 10 mM KCl, 2 mM MgCl_2_, 1 mM EDTA, 1 mM EGTA, 1 mM DTT and Protease inhibitor Cocktail (III)) on ice for 35 min and centrifuged at 720× *g* for five minutes. The pellet contained nuclei and the supernatant contained cytoplasmic proteins. To extract proteins from nuclear fraction, the pellet was resuspended with Buffer B (20 mM Tris pH 8.0, 100 mM NaCl, 2 mM, EDTA pH 8.0) with the addition of 300 mM NaCl and homogenized with 20 full strokes in Dounce on ice and centrifugated at 24,000× *g* for 20 min at 4 °C. Supernatant contained nuclear proteins.

### 4.8. Western Blot

Total protein was extracted using Sodium dodecyl sulfate (SDS) reducing buffer (Biorad, CA, USA) and separated on 8–12% gradient SDS polyacrylamide gels and transferred on Polyvinylidene difluoride (PVDF) membranes (Biorad, CA, USA). Membranes were blocked with 5% non-fat dry milk for 30 min at room temperature, followed by overnight incubation with a primary antibody against caspase-1 (1:300, Santa Cruze) at 4 °C. Membranes were washed with PBS with 0.1% Tween 20 and incubated for one hour at room temperature with a secondary antibody (goat polyclonal anti-mouse IgG, ab205719, 1:3000) for one hour. Membranes were stained using anti-beta-actin (1:4000, A01546, GenScript) antibody to normalize protein expression for total protein and cytoplasmic fraction. Membranes were stained with anti-lamin b1 (1:300, sc-20682, Santa Cruz) antibody and secondary human, bovine, horse, horseradish peroxidase-conjugated goat anti-mouse IgGs (H&L) (A106PS; American Qualex) to normalize protein expression for nuclear proteins. Western blot results were visualized using Clarity Western ECL reagents (Biorad, CA, USA) and a ChemiDoc XRS + (Biorad, CA, USA). 

### 4.9. Cytokine Assay

MILLIPLEX MAP Human Cytokine/Chemokine Magnetic Bead Panel-Premixed 41 Plex was used to analyze the cytokine secretion pattern of samples according to the manufacturer’s recommendations. Fifty microliters of the sample were used for cytokine analysis. The resulting data were analysed using the BioPlex 200 analyser with MasterPlex CT control software and MasterPlex QT analysis software (MiraiBio division of Hitachi Software, San Francisco, CA, USA). The results were presented as a heatmap graph produced using the web-based program Heatmapper (http://www.heatmapper.ca/) according to the method recommended by Babicki et al. [80]. 

### 4.10. Statistical Analysis

Statistical analysis was performed using SPSS 20 statistical software (IBM Corp., Armonk, NY, USA). One-way ANOVA, Tukey’s analyses and the independent sample t-test were performed to evaluate the findings of the real-time qPCR. The Kruskal–Wallis test was performed to evaluate ELISA. The data were presented as mean ± SE. Significance was established at a value of *p* < 0.05.

## Supplementary Materials

The following are available online, Table S1: Primer set sequences for the RT-qPCR analyze [81], Table S2: The effect of wild type and dominant negative Rab5, Rab7 and Rab11 plasmid transfection on changes in transcript levels of RAB5, RAB7 and RAB11 in HTC116 cells, Table S3: The effect of LPS/nigericin on NALP3 activation in HCT-116 cells. Table S4: The effect of DN Rab5 and WT Rab5 expressions on NALP3 activation, Table S5: The comparative effect of DN Rab5 and WT Rab5expression on mRNA regulation of NLRP3 inflammasome pathway, Table S6: The effect of RAB5 on IL-1β secretion in HCT116 cells, Table S7: The effect of DN Rab7 and WT Rab7 expressions on NALP3 activation, Table S8: The effect of RAB7 gene expression on mRNA regulation of NLRP3 inflammasome pathway, Table S9: The effect of RAB7 on IL-1β secretion in HCT116 cells, Table S10: The effect of DN Rab11 and WT Rab11 expressions on NALP3 activation, Table S11: The comparative effect of DN Rab11 and WT Rab11 on mRNA regulation of NLRP3 inflammasome pathway, Table S12: The effect of RAB11 on IL-1β secretion in HCT116 cells, Table S13: The effect of Rab5 on cytokine secretion levels in HCT116 cells (pg/mL).

## Abbreviations

ASC: an apoptosis-associated speck-like protein containing a caspase recruitment domain; NALP3: NACHT, LRR and PYD domains-containing protein 3; IL-1β: Interleukin 1 beta; CRC: Colorectal cancer; DN: Dominant negative, WT: Wild type, LPS: lipopolysaccharide; LN: LPS/Nigericin.
