# Supplementary material for: Rab GTPase Mediating Regulation of NALP3 in Colorectal Cancer
Source: Molecules. 2020 Oct 20;25(20):4834. doi: 10.3390/molecules25204834 (PMC7587934; doi:10.3390/molecules25204834)
Supplement: Supplementary file 1 [file molecules-25-04834-s001.zip › Supp. Tables-revised.docx]

**Supplementary Table S1:** Primer set sequences for the RT-qPCR analyze

| Primers | | |
| --- | --- | --- |
| *Pro-IL-1β* | F: | 5'-TCAGCACCTCTCAAGCAGAA-3' |
|  | R: | 5'-GGACTCTCTGGGTACAGCTC-3' |
| *Pro-CASP1* | F: | 5'-TGCCTTTCTTCTGGTCAGTG-3' |
|  | R: | 5'-TGCTGAGGTGAAGGAGAGAA-3' |
| *NLRP3* | F: | 5'-ATGAGTGCTGCTTCGACATC-3' |
|  | R: | 5'-TTGTCACTCAGGTCCAGCTC-3' |
| *RAB39A* | F: | 5'-TCTACCAGTTCCGCCTCATC-3' |
|  | R: | 5'-ATTGATCTGAACCGCTCCTG-3' |
| *RAB5* | F: | 5'- TTAGAAAAGCAGCCCCAATG-3' |
|  | R: | 5'- GTACTTCTGGGAGAGTCCGC-3' |
| *RAB7* | F: | 5'- CCTTCAGCAACACTTTCTTCCT-3' |
|  | R: | 5'- CCGTTTAGTCTCCTCCTCGG-3' |
| *RAB11* | F: | 5'- GCTCGGCCTCGACAAGTTC-3' |
|  | R: | 5'- ACTTATACCACTGCGTCTTCCT-3' |
| *ACTB* | F: | 5'-GACAGGATGCAGAAGGAGATTACT-3' |
|  | R: | 5'-TGATCCACATCTGCTGGAAGGT-3' |

Primary set sequences for *RAB5*, *RAB7* and *RAB11* were used according to study of da Silva et al. [81]. Primary set sequence for *Pro-IL-1β*, *Pro-CASP1*, *NLRP3* and *RAB39A* were design by GenScript DNA Sequencing Primers Design Tool (https://www.genscript.com/tools/dna-sequencing-primer-design).

**Supplementary Table S2.** The effect of wild type and dominant negative Rab5, Rab7 and Rab11 plasmid transfection on changes in transcript levels of RAB5, RAB7 and RAB11 in HTC116 cells

| mRNA |  | Std. E. | Mean Difference | P* | 95% CI | |
| --- | --- | --- | --- | --- | --- | --- |
|  |  |  |  |  | Lower | Upper |
| *RAB5* | Non-transfected /Rab5 DN | 4,13 | 0,50 | 0,992 | -12,18 | 13,18 |
|  | Non-transfected /Rab5 WT | 4,13 | -36,05 | **<0,001** | -48,73 | -23,37 |
|  | Rab5 DN /Rab5 WT | 4,13 | -36,55 | **<0,001** | -49,23 | -23,87 |
| *RAB7* | Non-transfected /Rab7 DN | 0,20 | 0,57 | 0,065 | -0,04 | 1,19 |
|  | Non-transfected /Rab7 WT | 0,20 | -1,06 | **0,005** | -1,67 | -0,44 |
|  | Rab7 DN /Rab7 WT | 0,20 | -1,63 | **<0,001** | -2,25 | -1,01 |
| *RAB11* | Non-transfected /Rab11 DN | 0,22 | 0,50 | 0,137 | -0,18 | 1,17 |
|  | Non-transfected /Rab11 WT | 0,22 | -3,07 | **<0,001** | -3,75 | -2,40 |
|  | Rab11 DN /Rab11 WT | 0,22 | -3,57 | **<0,001** | -4,25 | -2,90 |

*P value was calculated using One Way Anova and Tukey Test

**Supplementary Table S3.** The effect of LPS/Nigericin on NALP3 activation in HCT-116 cells.

|  |  |  |  | HCT-116 (non transfected) | | |  |  |
| --- | --- | --- | --- | --- | --- | --- | --- | --- |
| mRNA | U/LN | t | df | Mean Difference | Std. Error | P | %95 CI | |
|  |  |  |  |  |  |  | Lower | Upper |
| NLRP3 | U/LN | -28,80 | 4 | -11,39 | 0,03 | **<0,001** | -11,48 | -11,29 |
| Pro-CASP1 | U/LN | -9,60 | 4 | -6,27 | 0,64 | **0,001** | -8,06 | -4,47 |
| RAB39A | U/LN | -10,26 | 4 | -4,09 | 0,39 | **0,001** | -5,19 | -2,98 |
| Pro-IL1b | U/LN | -26,18 | 4 | -2,57 | 0,69 | 0,080 | -2,84 | -2,29 |

*P value was calculated using Independent Sample T Test

**Supplementary Table S4.** The effect of DN Rab5 and WT Rab5 expressions on NALP3 activation

| mRNA | Comparison |  |  | DN Rab5 | | |  |  |  |  | WT Rab5 | | |  |  |
| --- | --- | --- | --- | --- | --- | --- | --- | --- | --- | --- | --- | --- | --- | --- | --- |
|  |  | t | df | Mean Difference | Std. Error | P* | %95 CI | | t | df | Mean Difference | Std. Error | P* | %95 CI | |
|  |  |  |  |  |  |  | Lower | Upper |  |  |  |  |  | Lower | Upper |
| *NLRP3* | U/LN | -1,40 | 3 | -0,42 | 0,30 | 0,256 | -1,37 | 0,53 | -7,60 | 4 | -13,49 | 1,78 | **0,002** | -18,41 | -8,56 |
| *Pro-CASP1* | U/LN | -15,31 | 4 | -0,81 | 0,05 | **<0,001** | -0,95 | -0,66 | -15,40 | 3 | -23,43 | 0,09 | **<0,001** | -1,62 | -1,06 |
| *RAB39A* | U/LN | 0,85 | 4 | 0,37 | 0,44 | 0,441 | -0,84 | 1,59 | -22,61 | 2 | -6,16 | 0,27 | **0,002** | -7,33 | -4,98 |
| *IL-1β* | U/LN | 1,10 | 4 | 0,19 | 0,18 | 0,332 | -0,30 | 0,69 | -2,60 | 3 | -2,02 | 0,78 | 0,080 | -4,49 | 0,45 |

*P value was calculated using Independent Sample T test.

**Supplementary Table S5.** The comparative effect of DN Rab5 and WT Rab5 expression on mRNA regulation of NLRP3 inflammasome pathway

| mRNA | DN Rab5/ WT Rab5 | t | df | Mean Difference | Std. Error | P* | %95 CI | |
| --- | --- | --- | --- | --- | --- | --- | --- | --- |
|  |  |  |  |  |  |  | Lower | Upper |
| *NLRP3* | U | -2,18 | 4 | -1,09 | 0,50 | 0,095 | -2,47 | 0,30 |
|  | U/LN | -12,58 | 4 | -13,07 | 1,04 | **<0,001** | -15,96 | -10,18 |
| *Pro-CASP1* | U | -0,84 | 4 | -3,94 | 0,03 | 0,450 | -0,10 | 0,05 |
|  | U/LN | -8,92 | 4 | -22,62 | 0,06 | **<0,001** | -0,70 | -0,37 |
| *RAB39A* | U | 0,59 | 3 | 0,28 | 0,48 | 0,600 | -1,25 | 1,81 |
|  | U/LN | -21,91 | 4 | -6,53 | 0,30 | **<0,0001** | -7,36 | -5,70 |
| *IL-1β* | U | -1,17 | 4 | -0,20 | 0,17 | 0,306 | -0,66 | 0,27 |
|  | U/LN | -4,78 | 4 | -2,21 | 0,46 | **0,009** | -3,49 | -0,93 |

*P value calculated using Independent Samples T test

**Supplementary Table S6.** The effect of RAB5 on IL-1β secretion in HCT116 cells

|  | IL-1b | Mean Difference | P* |
| --- | --- | --- | --- |
|  |  |  |  |
| Non transfected HCT-116 | U/LN | -1,78 | **0,155** |
| DN Rab5 | U/LN | -1,78 | **0,002** |
| WT Rab5 | U/LN | -2,23 | **0,024** |
| DN Rab5/ WT Rab5 | U | -3,18 | **0,012** |
|  | U/LN | -0,45 | **<0,0001** |

*P value calculated using Kruskal-Wallis Tests

**Supplementary Table S7.** The effect of DN Rab7 and WT Rab7 expressions on NALP3 activation

|  |  |  |  | DN Rab7 | | |  |  |  |  | WT Rab7 | | |  |  |
| --- | --- | --- | --- | --- | --- | --- | --- | --- | --- | --- | --- | --- | --- | --- | --- |
| mRNA | Comparison | t | df | Mean Difference | Std. Error | P* | %95 CI | | t | df | Mean Difference | Std. Error | P* | %95 CI | |
|  |  |  |  |  |  |  | Lower | Upper |  |  |  |  |  | Lower | Upper |
| *NLRP3* | U/LN | 2,58 | 4 | 2,81 | 1,09 | 0,062 | -5,85 | 0,22 | -6,07 | 4 | -17,97 | 2,96 | **0,004** | -26,19 | -9,75 |
| *Pro-CASP1* | U/LN | -1,46 | 4 | -3,54 | 2,43 | 0,218 | -10,28 | 3,20 | -9,64 | 4 | -13,91 | 1,44 | **0,001** | -17,92 | -9,90 |
| *RAB39A* | U/LN | -3,81 | 4 | -1,43 | 1,43 | **0,019** | -9,39 | -1,47 | -3,54 | 4 | -20,85 | 1,25 | **0,024** | -71,08 | -8,62 |
| *IL-1β* | U/LN | -5,05 | 4 | -2,39 | 0,47 | **0,007** | -3,71 | -1,08 | -5,30 | 4 | -13,19 | 2,49 | **0,006** | -20,10 | -6,28 |

*P value calculated using Independent Sample T Tests

**Supplementary Table S8.** The effect of RAB7 gene expression on mRNA regulation of NLRP3 inflammasome pathway

| mRNA | DN Rab7/ WT Rab7 | t | df | Mean Difference | Std. Error | P* | %95 CI | |
| --- | --- | --- | --- | --- | --- | --- | --- | --- |
|  |  |  |  |  |  |  | Lower | Upper |
| *NLRP3* | U | -2,68 | 4 | -2,79 | 1,04 | 0,055 | -5,68 | 0,10 |
|  | U/LN | -11,41 | 4 | -20,78 | 1,82 | **<0,001** | -25,84 | -15,72 |
| *Pro-CASP1* | U | 1,27 | 4 | 0,92 | 0,73 | 0,275 | -1,10 | 2,95 |
|  | U/LN | -6,37 | 4 | -10,37 | 1,63 | **0,003** | -14,89 | -5,85 |
| *RAB39A* | U | 0,91 | 4 | 1,23 | 0,45 | 0,414 | -4,58 | 9,04 |
|  | U/LN | -5,26 | 4 | -19,42 | 6,55 | **0,006** | -52,60 | -16,24 |
| *IL-1β* | U | -1,91 | 4 | -0,50 | 0,26 | 0,128 | -1,22 | 0,22 |
|  | U/LN | -7,38 | 4 | -10,80 | 1,46 | **0,002** | -14,86 | -6,73 |

*P value calculated using Independent Samples T Test

**Supplementary Table S9.** The effect of RAB7 on IL-1β secretion in HCT116 cells

|  | IL-1β | Mean Difference | P* |
| --- | --- | --- | --- |
|  |  |  |  |
| DN Rab7 | U/LN | -1,78 | **0,021** |
| WT Rab7 | U/LN | -2,30 | **0,003** |
| DN Rab7/ WT Rab7 | U | -1,58 | 0,089 |
|  | U/LN | -0,52 | 0,187 |

*P value calculated using Kruskal-Wallis Test

**Supplementary Table S10.** The effect of DN Rab11 and WT Rab11 expressions on NALP3 activation

|  |  |  |  | DN Rab11 | | |  |  |  |  | WT Rab11 | | |  |  |
| --- | --- | --- | --- | --- | --- | --- | --- | --- | --- | --- | --- | --- | --- | --- | --- |
| mRNA | Comparison | t | df | Mean Difference | Std. Error | P | %95 CI | | t | df | Mean Difference | Std. Error | P | %95 CI | |
|  |  |  |  |  |  |  | Lower | Upper |  |  |  |  |  | Lower | Upper |
| *NLRP3* | U/LN | -4,92 | 4 | -11,06 | 2,25 | **0,008** | -17,31 | -4,82 | -28,87 | 4 | -11,97 | 0,41 | **<0,0001** | -13,12 | -10,82 |
| *Pro-CASP1* | U/LN | -29,25 | 4 | -6,62 | 0,06 | **<0,0001** | -1,77 | -1,47 | -2,99 | 4 | -6,95 | 0,32 | **0,041** | -1,83 | -0,07 |
| *RAB39A* | U/LN | -82,60 | 3 | -4,31 | 0,05 | **<0,0001** | -4,48 | -4,14 | -4,84 | 4 | -3,17 | 0,66 | **0,008** | -4,99 | -1,35 |
| *IL-1β* | U/LN | -3,64 | 3 | -0,56 | 0,15 | **0,036** | -1,06 | -0,07 | -3,87 | 4 | -4,10 | 1,06 | **0,018** | -7,04 | -1,16 |

*P value calculated using Independent Samples T Test

**Supplementary Table S11.** The comparative effect of DN Rab11 and WT Rab11 on mRNA regulation of NLRP3 inflammasome pathway

| mRNA | DN Rab11/ WT Rab11 | t | df | Mean Difference | Std. Error | P* | %95 CI | |
| --- | --- | --- | --- | --- | --- | --- | --- | --- |
|  |  |  |  |  |  |  | Lower | Upper |
| *NLRP3* | U | -9,55 | 4 | -1,32 | 0,14 | **0,001** | -1,71 | -0,94 |
|  | U/LN | -0,69 | 4 | -0,91 | 1,32 | 0,529 | -4,58 | 2,76 |
| *Pro-CASP1* | U | -9,96 | 4 | -1,16 | 0,12 | **0,001** | -1,49 | -0,84 |
|  | U/LN | 3,64 | 4 | 0,32 | 0,19 | **0,022** | 0,16 | 1,19 |
| *RAB39A* | U | -5,11 | 3 | -0,95 | 0,19 | **0,015** | -1,54 | -0,36 |
|  | U/LN | 3,00 | 4 | 1,14 | 0,38 | **0,040** | 0,08 | 2,19 |
| *IL-1β* | U | -7,47 | 4 | -1,28 | 0,17 | **0,002** | -1,75 | -0,80 |
|  | U/LN | -5,72 | 4 | -3,54 | 0,62 | **0,005** | -5,25 | -1,82 |

*P value calculated using Independent Samples T Test

**Supplementary Table S12.** The effect of RAB11 on IL-1β secretion in HCT116 cells

|  | IL-1b | Mean Difference | P |
| --- | --- | --- | --- |
|  |  |  |  |
| DN Rab11 | U/LN | -0,67 | 0,062 |
| WT Rab11 | U/LN | -1,71 | 0,403 |
| DN Rab11/ WT Rab11 | U | -0,80 | 0,130 |
|  | U/LN | -1,04 | 0,338 |

*P value calculated using Kruskal-Wallis Test

**Supplementary Table S13.** The effect of Rab5 on cytokine secretion levels in HCT116 cells (pg/mL)

| Cytokine | U | | LN | | Cytokine | U | | LN | |
| --- | --- | --- | --- | --- | --- | --- | --- | --- | --- |
|  | Rab5 DN | Rab5 WT | Rab5 DN | Rab5 WT |  | Rab5 DN | Rab5 WT | Rab5 DN | Rab5 WT |
| EGF | 2,2 | 6,7 | 3,67 | 14,02 | IL-17A | 2,02 | 2,02 | 2,02 | 2,02 |
| FGF-2 | 2,84 | 2,84 | 2,84 | 4,87 | IL1Ra | 1,72 | 1,72 | 1,72 | 1,72 |
| Eotaxin | 2,22 | 2,22 | 2,22 | 2,22 | IL1a | 1,62 | 1,62 | 1,62 | 1,62 |
| TGF-a | 5,22 | 14,15 | 24,35 | 27,84 | IL-9 | 2,11 | 18,81 | 12,47 | 61,71 |
| G-CSF | 1,69 | 71,87 | 29,16 | 312,17 | IL-1b | 8,95 | 12,13 | 10,73 | 14,36 |
| Flt-3L | 1,76 | 1,76 | 1,76 | 1,76 | IL-2 | 2,01 | 2,01 | 2,01 | 2,01 |
| GM-CSF | 9,13 | 16,03 | 9,13 | 12,81 | IL-3 | 1,88 | 1,88 | 1,88 | 1,88 |
| Fractalkine | 2,09 | 405,64 | 74,66 | 270,82 | IL-4 | 2,06 | 2,06 | 2,06 | 2,06 |
| IFNa2 | 6,25 | 45,02 | 1,54 | 6,25 | IL-5 | 1,61 | 1,61 | 1,61 | 1,61 |
| IFN-y | 1,66 | 1,66 | 1,66 | 1,66 | IL-6 | 1,94 | 7,15 | 116,66 | 163,94 |
| GRO | 60,88 | 172,22 | 527,09 | 289,26 | IL-7 | 2,19 | 2,19 | 2,19 | 2,19 |
| IL-10 | 1,06 | 1,06 | 1,06 | 1,06 | IL-8 | 146,87 | 528,32 | 1114 | 519,21 |
| MCP-3 | 1,86 | 1,86 | 1,86 | 1,86 | IP10 | 41,37 | 158,7 | 26,43 | 43,59 |
| IL12p40 | 2,14 | 2,14 | 2,14 | 2,14 | CCL-2 | 884,42 | 3349 | 962,49 | 808,68 |
| MDC | 31,39 | 78,16 | 3,21 | 9,8 | MIP-1a | 1,96 | 1,96 | 1,96 | 1,96 |
| IL12p70 | 1,48 | 1,48 | 1,48 | 1,48 | MIP-1b | 1,72 | 1,72 | 1,72 | 1,72 |
| PDGF-AA | 106,14 | 183,52 | 16,22 | 32,87 | Rantes | 2,71 | 2,71 | 2,71 | 2,71 |
| IL-13 | 1,2 | 1,2 | 1,2 | 1,2 | TNFa | 2 | 10,74 | 18,71 | 41,32 |
| PDGF-AB/BB | 1,66 | 498,26 | 1,66 | 220,43 | TNF-b | 1,69 | 1,69 | 1,69 | 1,69 |
| IL-15 | 1,73 | 1,73 | 1,73 | 1,73 | VEGF | 243,53 | 690,72 | 38,48 | 73,76 |
| sCD40L | 1,67 | 1,67 | 1,67 | 1,67 |  |  |  |  |  |
