# Supplementary material for: Rab GTPase Mediating Regulation of NALP3 in Colorectal Cancer
Source: Molecules. 2020 Oct 20;25(20):4834. doi: 10.3390/molecules25204834 (PMC7587934; doi:10.3390/molecules25204834)

Caspase 1 and β-actin immunoblotted membranes (Total protein). **A**: Pro-caspase-1, Caspase-1, **B**: β-actin, **C**: Marker

A

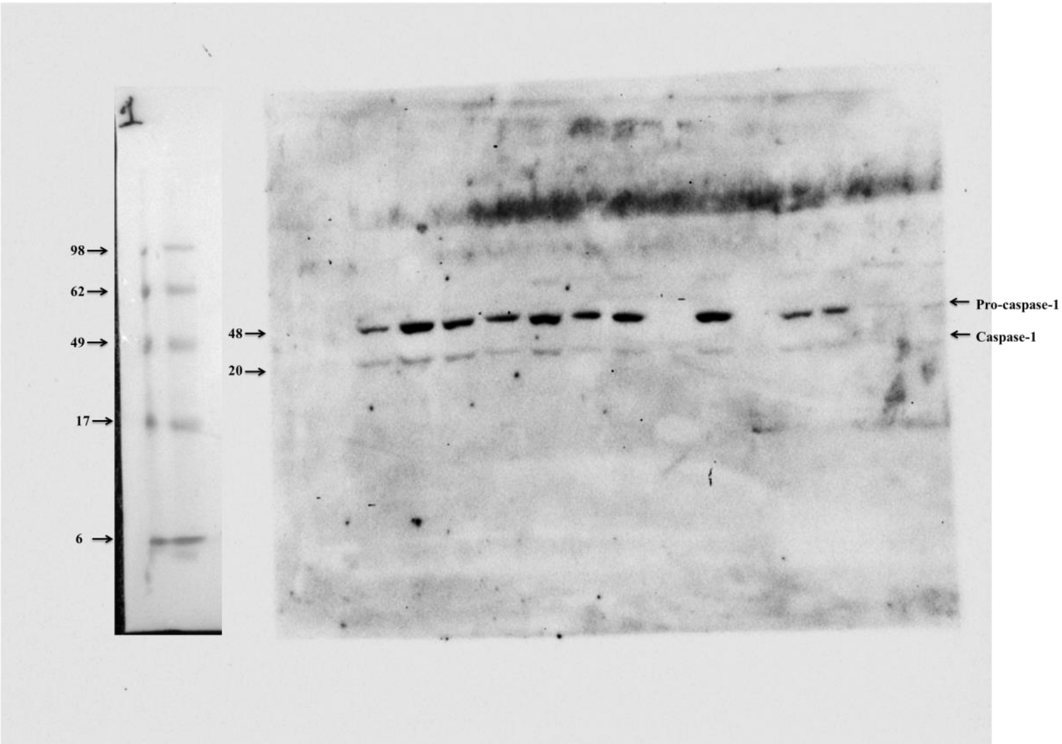

B

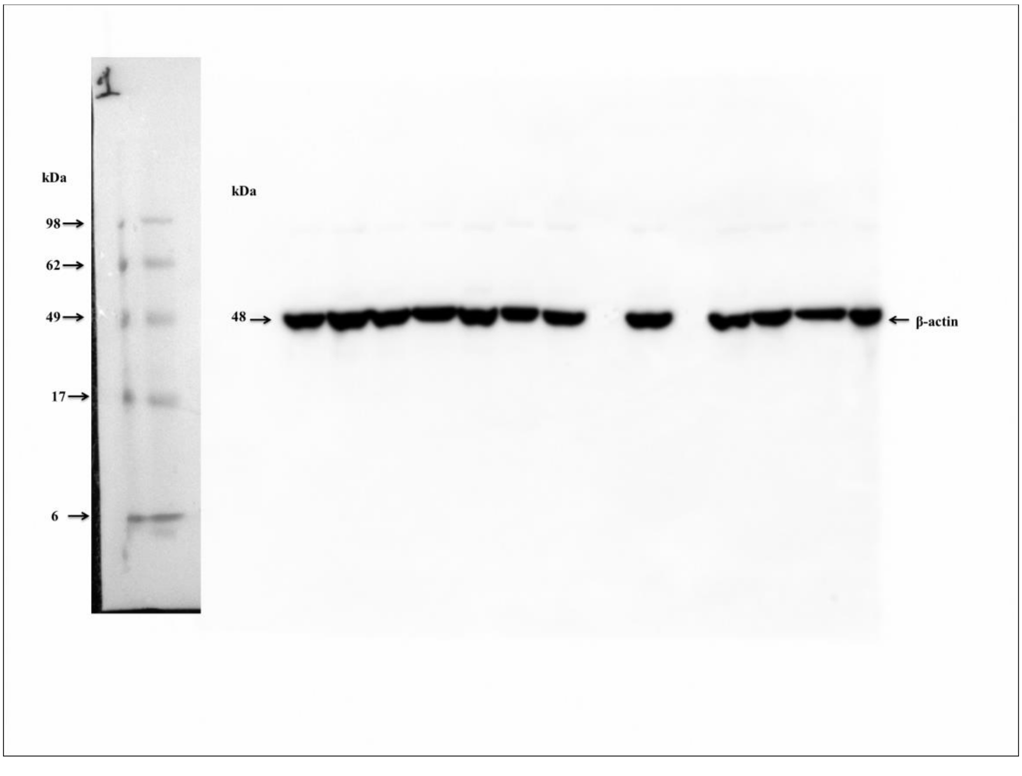

C

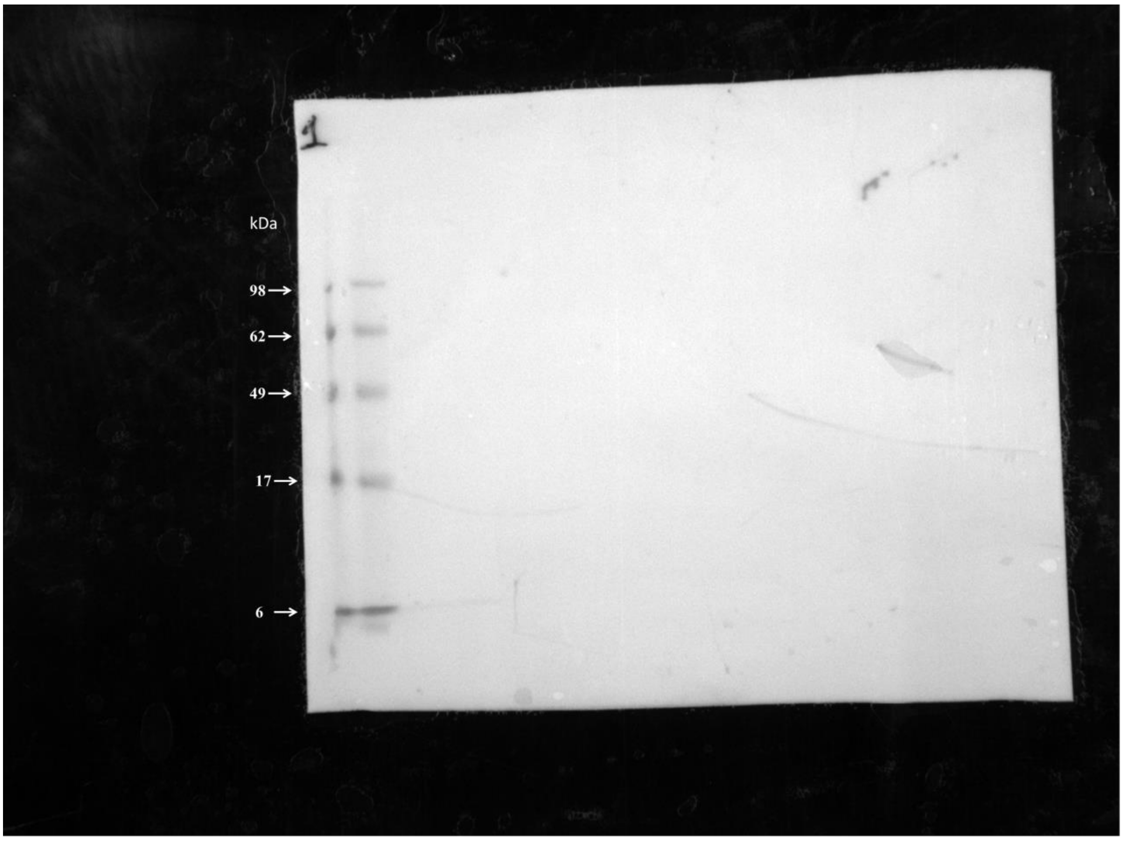

Caspase 1 and β-actin immunoblotted membranes in cytoplasmic fraction of wild type plasmid transfected HCT-116 cells. **D**: Pro-caspase-1, Caspase-1, **E**: β-actin, **F**: Marker

D

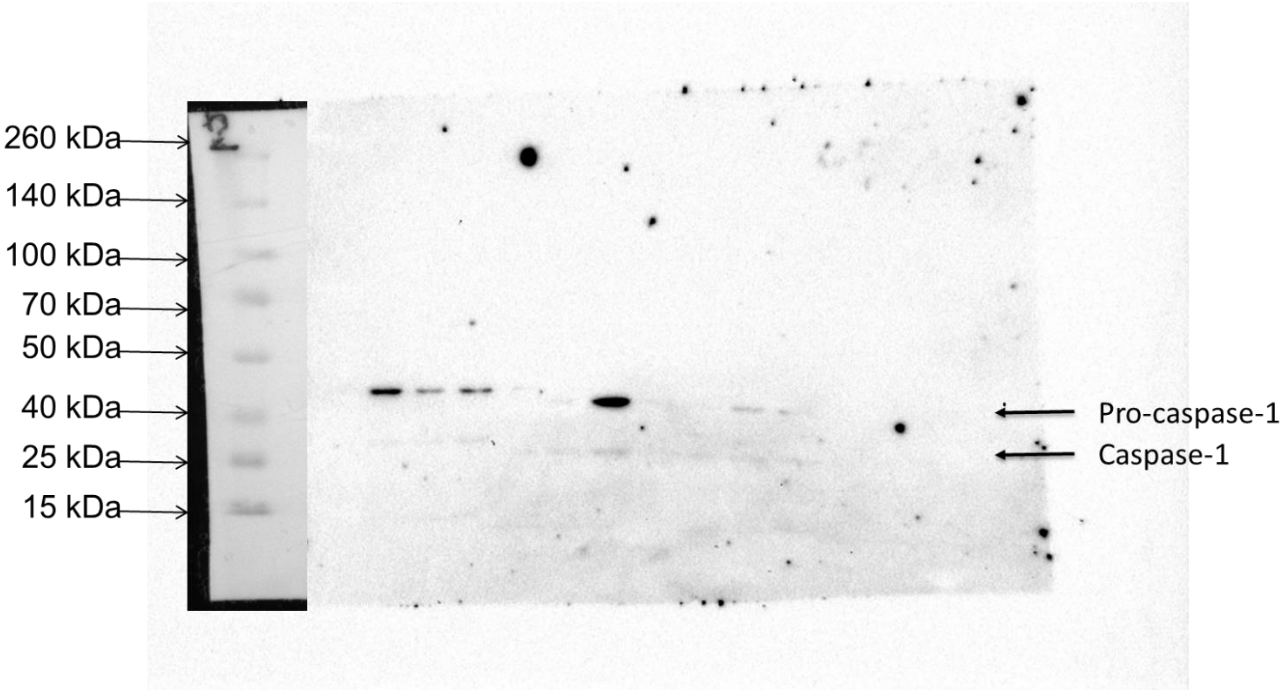

E

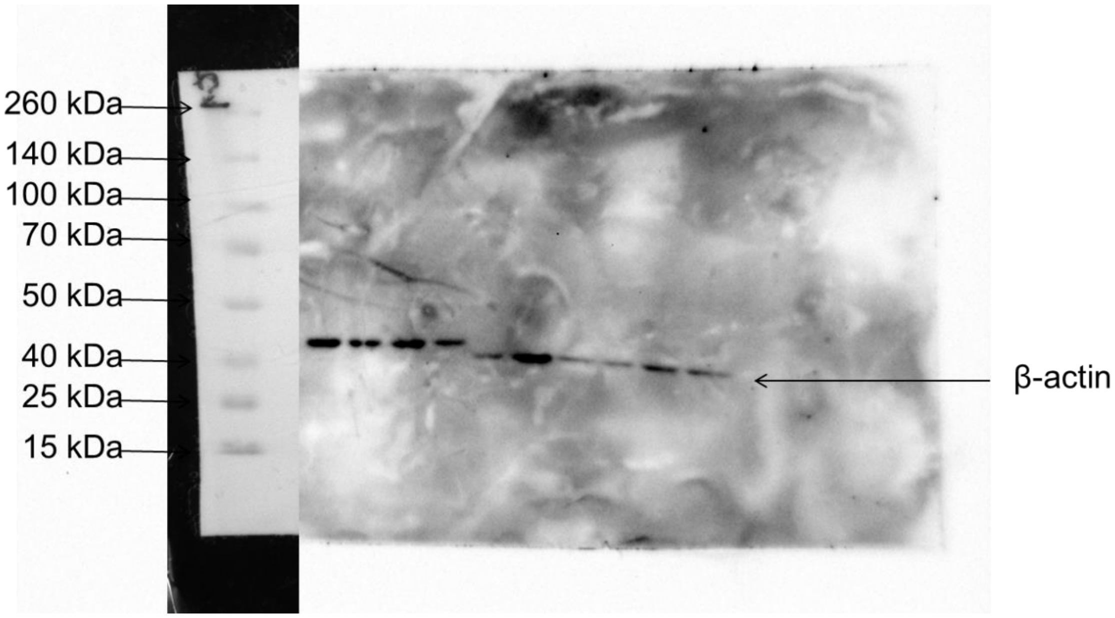

F

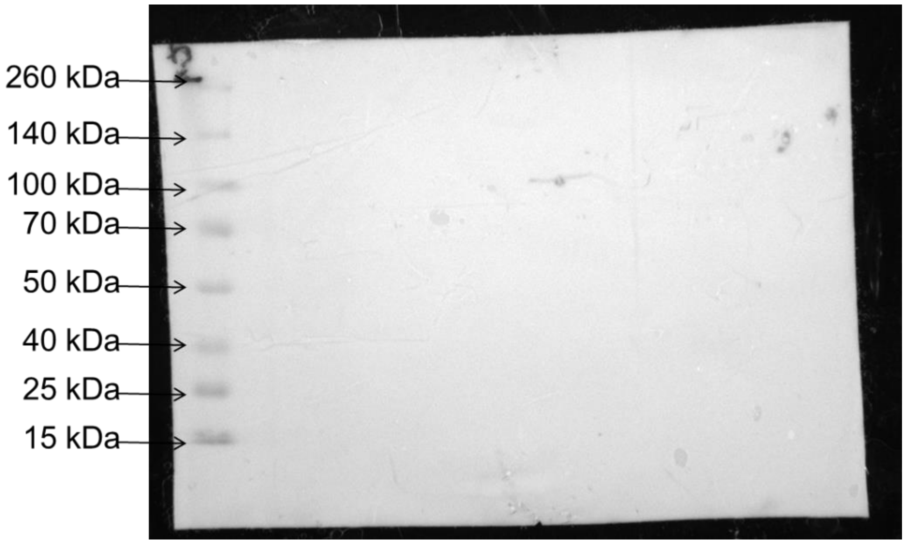

Caspase 1 and β-actin immunoblotted membranes in cytoplasmic fraction of dominant-negative plasmid transfected HCT-116 cells. **G**: Pro-caspase-1, Caspase-1, **H**: β-actin, **I**: Marker

G

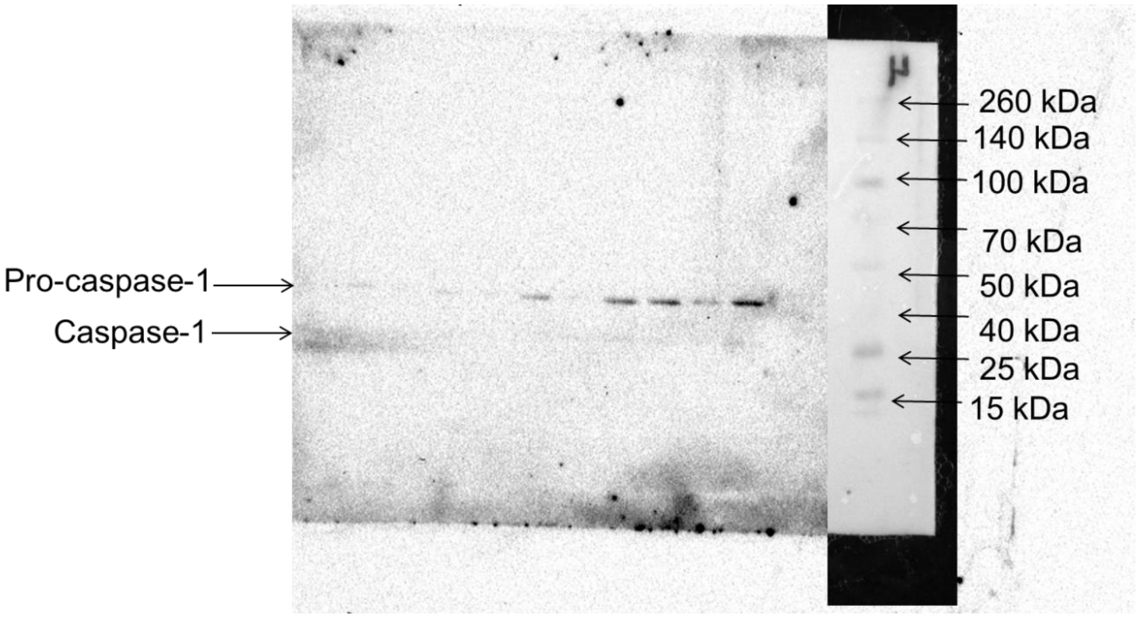

H

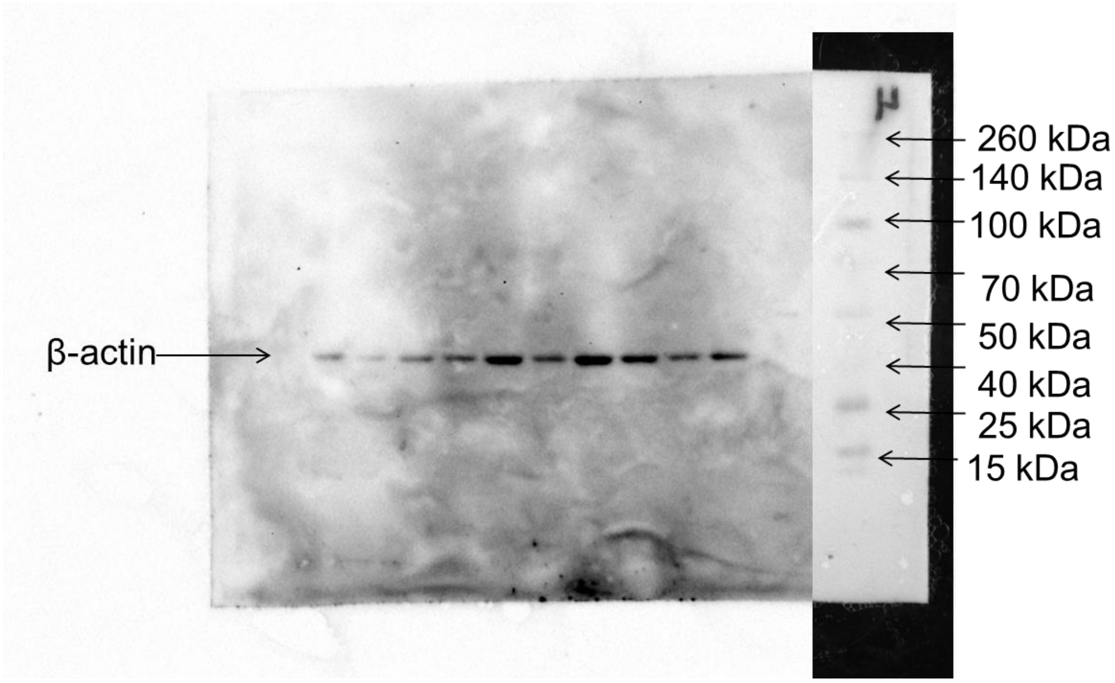
β-actin
260 kDa
140 kDa
100 kDa
70 kDa
50 kDa
40 kDa
25 kDa
15 kDa

I

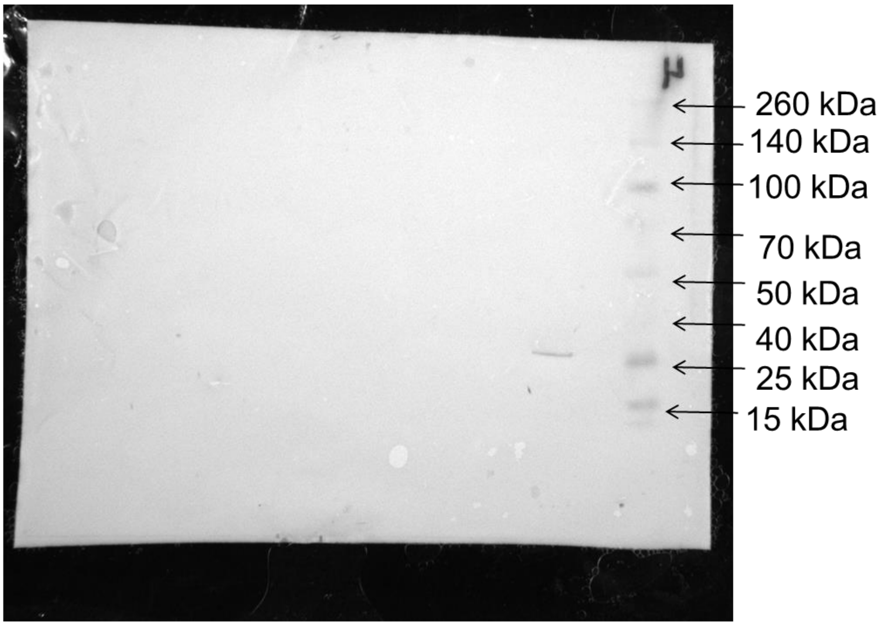
260 kDa
140 kDa
100 kDa
70 kDa
50 kDa
40 kDa
25 kDa
15 kDa

J: Caspase 1 and Lamin B1 immunoblotted membranes in nuclear fraction of plasmid transfected HCT-116 cells

Caspase-1

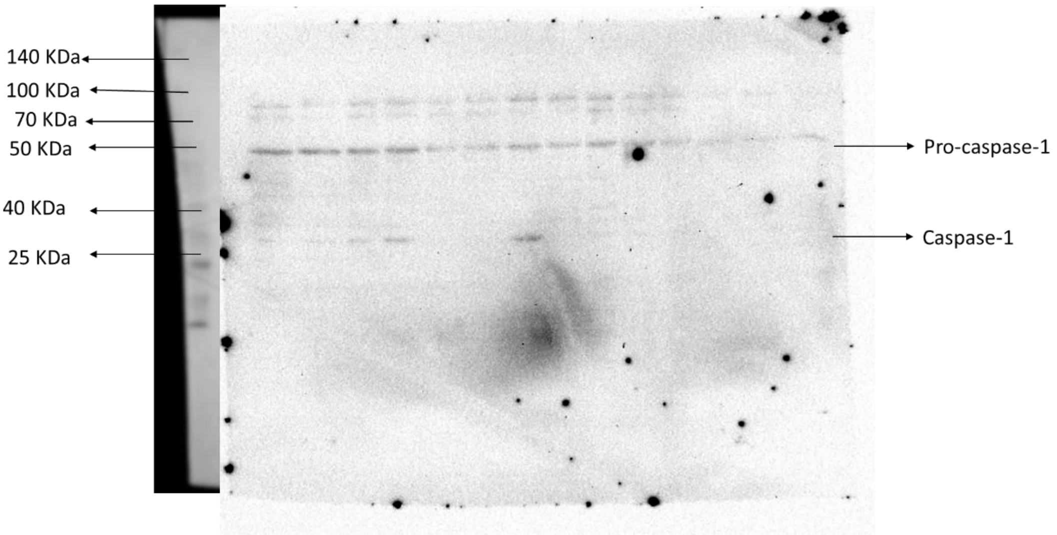

Lamin B1

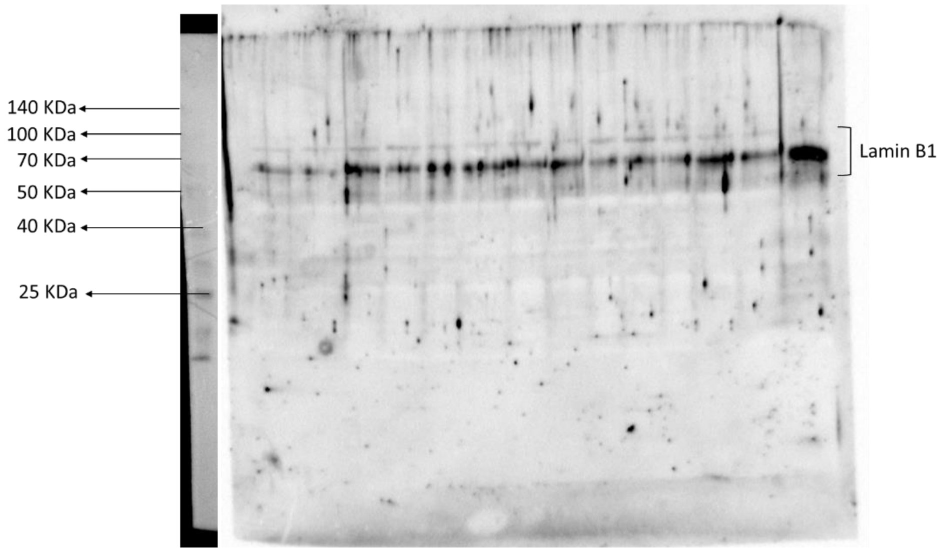

L: Protein marker for nuclear fraction

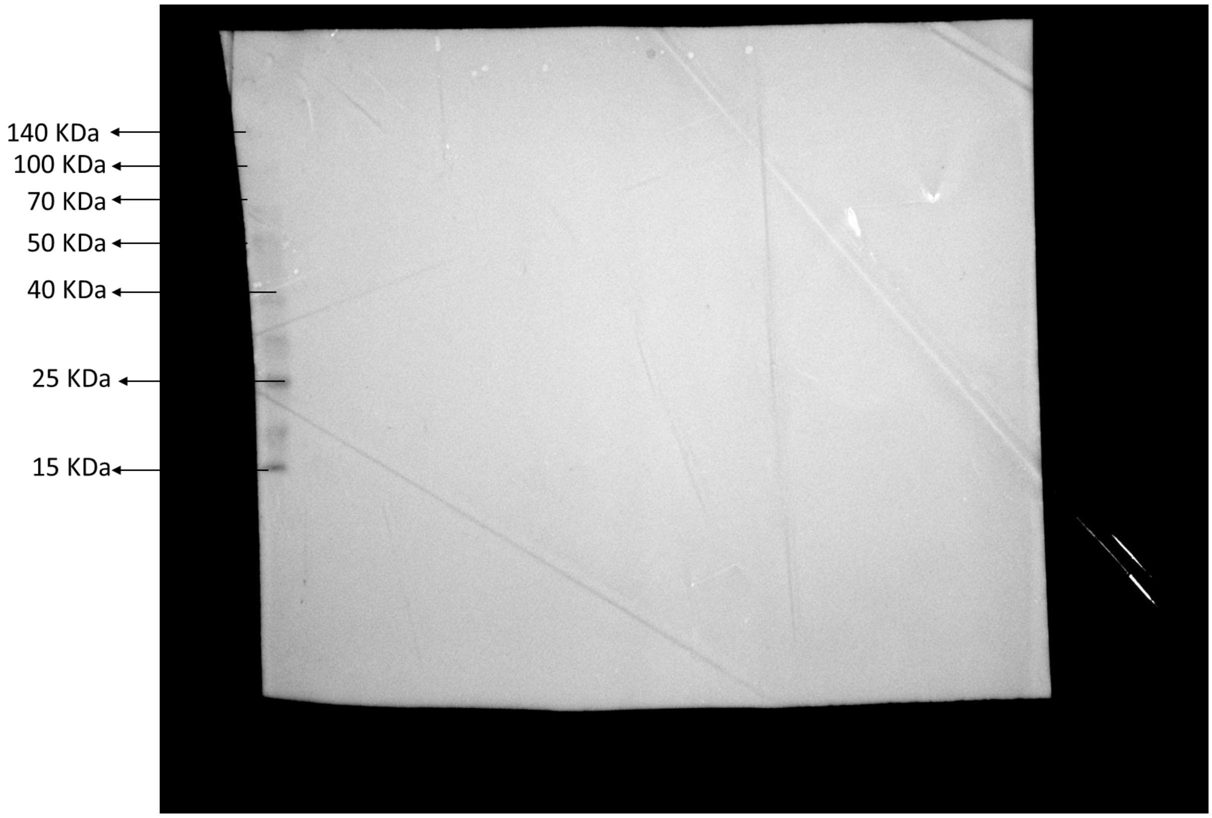

Supplement: Supplementary file 1 [file molecules-25-04834-s001.zip › Original membranes-supp file.pdf]
